# Emergence of CpG-cluster blanket methylation in aged tissues: a novel signature of epigenomic aging

**DOI:** 10.1093/nar/gkaf354

**Published:** 2025-05-10

**Authors:** Yong-Kook Kang, Byungkuk Min, Jaemin Eom, Jung Sun Park, Jaewoong Jang, Sangkyun Jeong

**Affiliations:** Aging Convergence Research Center (ACRC), Development and Differentiation Research Center, Korea Research Institute of Bioscience Biotechnology (KRIBB), 125 Gwahak-ro, Yuseong-gu, Daejeon 34141, South Korea; Department of Functional Genomics, University of Science and Technology (UST), 217 Gajeong-ro, Yuseong-gu, Daejeon 34113, South Korea; Aging Convergence Research Center (ACRC), Development and Differentiation Research Center, Korea Research Institute of Bioscience Biotechnology (KRIBB), 125 Gwahak-ro, Yuseong-gu, Daejeon 34141, South Korea; Aging Convergence Research Center (ACRC), Development and Differentiation Research Center, Korea Research Institute of Bioscience Biotechnology (KRIBB), 125 Gwahak-ro, Yuseong-gu, Daejeon 34141, South Korea; Department of Functional Genomics, University of Science and Technology (UST), 217 Gajeong-ro, Yuseong-gu, Daejeon 34113, South Korea; Aging Convergence Research Center (ACRC), Development and Differentiation Research Center, Korea Research Institute of Bioscience Biotechnology (KRIBB), 125 Gwahak-ro, Yuseong-gu, Daejeon 34141, South Korea; Aging Convergence Research Center (ACRC), Development and Differentiation Research Center, Korea Research Institute of Bioscience Biotechnology (KRIBB), 125 Gwahak-ro, Yuseong-gu, Daejeon 34141, South Korea; Genomics Department, Keyomics Co. Ltd, 17 Techno4-ro, Yuseong-gu, Daejeon 34013, South Korea

## Abstract

Aging is accompanied by widespread DNA methylation changes across the genome. While age-related methylation studies typically focus on individual CpGs, cluster analysis provides more robust data and improved interpretation. We characterized age-associated CpG-cluster methylation changes in mouse spleens, peripheral blood mononuclear cells, and livers. We identified a novel signature termed blanket methylations (BMs), fully methylated CpG clusters absent in young tissues but appearing in aged tissues. BM formation was locus- and cell-dependent, with minimal overlap among tissues. Statistical analysis, heterogeneity assessment, and random modeling demonstrated that BMs arise through nonrandom mechanisms and correlate with accelerated aging. Notably, BMs appeared in chronologically young mice with progeroid or disease-driven aging, including in 4-month-old *Zmpste24^−/−^* (lifespan ∼5 months) and 3-month-old Huntington’s disease model mice (lifespan ∼4 months). The detection of BMs in purified CD4+ T cells demonstrated that their occurrence is intrinsic to aging cells rather than a result of infiltration from other tissues. Further investigation revealed age-related downregulation of zinc-finger-CxxC-domain genes, including *Tet1* and *Tet3*, which protect CpG islands from methylation. Importantly, TET1 or TET3 depletion induced BM formation, linking their loss to age-associated methylation drift. These findings establish BMs as a robust marker of epigenomic aging, providing insight into age-related methylation changes.

## Graphical Abstract

**Graphical Abstract:**
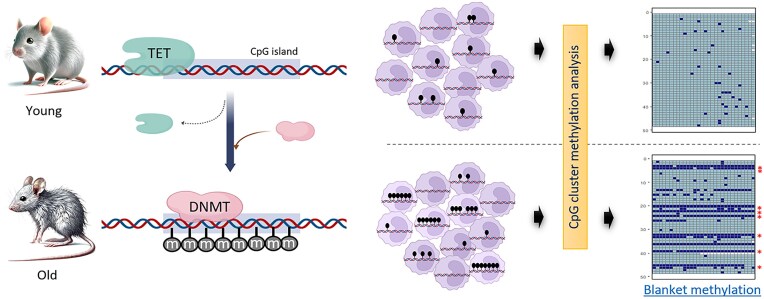

## Introduction

DNA methylation, predominantly occurring at palindromic CpG dinucleotides, represents a stable epigenetic mark faithfully propagated during DNA replication [1]. This symmetry in methylation across DNA strands ensures the precise transmission of methylation patterns from parent to daughter strands, establishing a form of cellular memory. As a prototype mechanism of epigenetic inheritance, DNA methylation serves as a dynamic substrate for epigenetic regulation, responding to cellular and environmental cues [2, 3]. These methylation changes are fundamental to a variety of biological processes, including gene regulation, cell differentiation [4, 5], genomic imprinting [6], silencing of transposable elements [7], and X-chromosome inactivation [8]. During early development, DNA methylation patterns undergo extensive remodeling, transitioning from pluripotent stem cells to lineage-specific somatic cells [5]. Once established, these patterns are typically maintained with high fidelity in somatic cells, contributing to cellular identity [9]. However, in adult somatic tissues, DNA methylation patterns are increasingly influenced by environmental factors and stochastic events, leading to a gradual erosion of methylation states [10]. Unlike the tightly regulated methylation changes during development, these late-life alterations are less controlled and may reflect an age-associated decline in epigenetic stability [11]. Such age-related changes in DNA methylation have been implicated in various pathologies, underscoring their potential significance in understanding aging and disease.

Recent advances in massively parallel sequencing (MPS) technology have enabled detailed investigations into age-related global methylation changes, offering unprecedented resolution across the genome. These studies revealed that DNA methylation patterns undergo significant remodeling during aging, characterized by global and site-specific modifications that correlate with physiological decline and molecular damage. For instance, Sziraki *et al.*, in their analysis of blood samples from 141 mice (3–35 months of age) using reduced representation bisulfite sequencing (RRBS), observed that aging leads to global remodeling of the DNA methylome, increased entropy, and site-specific methylation shifts, with calorie restriction mitigating these changes [12]. Similarly, Lu *et al.* found in a study utilizing DNA methylation profiles from multiple tissues (11 754 samples covering 59 tissue types across 185 mammalian species) that age-related methylation changes are conserved across mammals, linking these changes to evolutionary processes and underscoring universal mechanisms of aging [13]. Olecka *et al.*, examining colon samples from 83 male mice (3–28 months of age) using RRBS, identified nonlinear methylation changes during early-to-mid and mid-to-late life transitions, highlighting DNA methylation as a potential tool for staging biological aging [14, 15]. Bacalini *et al.*, using the Infinium 450K BeadChip array to analyze blood samples from 752 individuals aged from newborn to nonagenarian, identified differentially methylated regions consistently associated with aging, emphasizing their potential as biomarkers for biological age [16]. Collectively, these findings demonstrate that aging is universally associated with pervasive alterations in DNA methylation patterns across the genome. Importantly, genomic regions with low initial methylation levels tend to gain methylation (hypermethylation) with age, while highly methylated regions tend to lose methylation (hypomethylation) [17]. This phenomenon, known as epigenetic drift, leads to a “regression toward the mean” pattern of methylation change [18, 19], highlighting the region-specific nature of these modifications during aging.

The precise mechanisms underlying these deviations from established DNA methylation patterns remain unclear. Several epigenetic modifiers, including DNA methyltransferases (DNMTs), sirtuins, ten-eleven translocation (TET) family of dioxygenases, and methyl-binding domain proteins have been proposed as key regulators of these changes [20–26]. Additionally, shifts in cellular composition within tissues, such as changes in the proportions of white blood cells, may contribute to the observed alterations in DNA methylation patterns with age [27, 28]. Among these regulators, TET proteins play a pivotal role in shaping DNA methylation patterns by oxidizing 5-methylcytosine to 5-hydroxymethylcytosine (5hmC) [29], thereby facilitating both active and passive DNA demethylation [30]. In mammals, there are three TET proteins: TET1, TET2, and TET3. TET1 and TET3 share a structurally similar N-terminal CXXC DNA-binding domain, which enables them to bind CpG-rich regions, particularly CpG islands (CGIs) at gene promoters [31, 32]. In contrast, TET2 lacks this domain and is primarily located in gene bodies and exons [33]. TET1, notably enriched at CGIs and active promoters, exhibits binding that strongly correlates with CpG density and histone modification marks such as H3K4 trimethylation [34, 35]. These localized binding preferences allow TET proteins to regulate DNA demethylation and transcriptional activation at specific genomic regions. Their recruitment is further influenced by interactions with transcription factors, including NANOG [36], PRDM14 [37], PRC2 [38], WT1 [39], PU.1 [40], and CTCF [41]. TET proteins also play critical roles in aging and age-related diseases. Studies indicate that both TET expression and 5hmc levels decline with age in various tissues and cells in humans and mice [42–45]. This reduction is associated with widespread DNA demethylation, contributing to the epigenetic drift observed during aging. Moreover, impaired TET function has been implicated in neurodegenerative diseases such as Alzheimer’s disease, where age-associated TET decline exacerbates pathological processes [46–48]. Despite growing recognition of their roles, the precise contributions of TET proteins to global DNA demethylation during aging remain incompletely understood.

To gain insight into the mechanisms underlying epigenetic drift and to identify the molecular features that promote these age-associated changes, it may be valuable to investigate the extent of regional randomness of age-associated methylation changes. The age-related methylation changes may occur in a spatially clustered manner, reflecting regional regulation, or as spurious occurrences at single CpG sites, which are less likely to be functionally significant. The former suggests that investigating age-related CpG clusters with respect to chromatin configuration and DNA sequences could uncover specific epigenetic modifiers and transcription factors involved in these changes. Many studies have typically focused on singleton CpG sites in the aging epigenetics field. While this approach has provided valuable insights, shifting the focus to CpG clusters may yield a more reliable and biologically meaningful understanding of methylation changes, enhance signal detection, and emphasize functional relevance.

Age-related gain of DNA methylation, or hypermethylation, commonly occurs in gene promoters, particularly at promoter-associated CGIs, potentially altering the transcriptional activity of associated genes [24, 49]. These changes may result from *de novo* methylation of singleton CpGs or the emergence of highly methylated alleles from a subset of cells with varying characteristics, such as those undergoing fast senescence. By employing DNA methylation phasing, which identifies the positional relationship between methylated and unmethylated CpGs along DNA molecules, this study aims to provide a comprehensive understanding of these patterns. Such efforts will help elucidate the epigenetic mechanisms underlying aging and age-related diseases, ultimately facilitating the development of effective diagnostic and therapeutic strategies.

## Materials and methods

### Mouse and nucleic acid preparation

If not stated otherwise, for methylation analysis, C57BL6/J mice of different ages were purchased from the Korea Research Institute of Bioscience and Biotechnology (KRIBB). Additionally, R6/2 mice (B6CBA-R6/2; JAX stock #002810; [50]), which have 160 ± 5 CAG repeat expansion in the exon-1 of the Huntington disease (HD) gene, were purchased from the Jackson Laboratory. Information on YAC128 HD model mice was described elsewhere [51]. Mice were sacrificed at the designated ages for tissue collection following approval from the Institutional Animal Care and Use Committee. Frozen liver tissues from 4-month-old *Zmpste24^−/−^* mice were generously provided by Prof. Youngsam Lee at DGIST, South Korea. Genomic DNAs were extracted from the livers and peripheral blood mononuclear cells (PBMCs) using All Prep DNA Mini Kit (Qiagen) following the manufacturer’s instructions.

### Ethics approval and consent to participate

All experimental and research procedures were approved by and in accordance with relevant guidelines and regulations.

### RRBS and sequence analysis

Bisulfite-converted *Msp*I-restricted genomic fragments were molecularly indexed and sequenced using an in-house developed RRBS method as previously reported [17]. Briefly, *Msp*I-fragmented genomic DNAs (100 ng) were purified using a Zymo DNA Clean-up Kit (Zymo Research) and ligated with the adaptors (2 pmol/each) at 25°C for 30 min. Following the manufacturer’s instructions, bisulfite conversion was performed for the adaptor-attached MspI fragments using the EZ DNA Methylation-Gold™ Kit (Zymo Research). After bisulfite conversion, MspI fragments were subsequently amplified by two successive rounds of polymerase chain reaction (PCR) to generate sequencing-ready libraries. MPS was performed on the Illumina Hiseq platform with 151-bp paired-end reading. To obtain singleton CpG methylation (scMet) levels, we followed the protocol previously reported [52]. Briefly, the reads from the RRBS sequencing were processed using trim_galore (version 0.6.6) to remove the adaptors. The trimmed reads were then aligned to the mm10 mouse reference genome using the Bismark package (version 0.22.3). The methylation status of each CpG site was decided using *ad hoc* Perl scripts and clonal copies, which were identified by the presence of the same molecular barcode, were removed by the deduplication process. The scMet levels were collected, and only those with at least 10 deduplicated reads with unique molecular barcodes in each sample were used for subsequent analysis.

### Computation of cluster CpG methylation levels

To obtain cluster CpG methylation (ccMet) levels, raw RRBS reads were preprocessed to remove Illumina adapter sequences and low-quality bases by trim_galore v0.6.8dev. Next, paired-end reads were merged into single reads by FLASH 1.2.11 (Fast Length Adjustment of SHort reads; [53]) to eliminate the chance of double counting and increase the reliability of fragment-wise DNA methylation patterns. Additionally, three bases at the 5′ end and two bases at the 3′ end of each merged read were clipped off to remove *Msp*I-digested sites. Single-end mapping was performed by Bismark v0.23.1dev to generate BAM files for each sample. From the resulting BAM file, coordinate information for each read (RNAME for chromosome, POS for read start position, and POS + length of the read for end position) and its methylation calls (XM-tag) were extracted. For the calculation of fragment-wise DNA methylation levels, methylation calls specific to the CpG context were extracted and scored as “0” for an unmethylated cytosine (z) and “1” for a methylated cytosine (Z). Finally, we summed up the scores in each read to calculate the methylation level. For more consistent outcomes, we combined all the data in triplicates for each age group. Principal component analysis (PCA) was performed to visualize and assess the variability in scMet and ccMet levels across different samples. Following the standardization of methylation levels among samples, PCA was conducted using the prcomp () function in R with centered and scaled data to ensure comparability. The first two principal components (PC1 and PC2) were extracted to capture the major sources of variance. PCA results were visualized as a scatter plot, with samples color-coded according to age groups. All statistical analyses and visualizations were conducted in R (versio 4.1.2) using the ggplot2 and factoextra packages.

When comparing the scMet and ccMet metrics, it is important to note their respective dependencies and strengths. For scMet, while it is influenced by read depth, our calculations are designed to ensure that adequate coverage thresholds are met, thereby minimizing variability caused by differences in sequencing depth. For ccMet, we acknowledge that its value depends on the number of CpGs within a *Msp*I fragment. However, this characteristic aligns with its purpose of capturing methylation density over biologically relevant CpG clusters, rather than restricting measurements to fixed intervals. We believe that incorporating all CpGs within a fragment provides a more accurate representation of the natural genomic architecture compared with using artificially fixed window sizes.

### Generation of random model of DNA methylations

To construct a randomly methylated “old” model, we isolated reads containing a specified number of CpGs or more and calculated ccMet levels for individual MspI loci in both young and old samples. The differences in ccMet levels between the young and old samples were then determined. Based on the number of reads and ccMet values for each MspI locus, we generated a matrix representing the old samples, maintaining the same fraction of methylated cytosines and the same number of reads but with the methylation randomly distributed. To achieve this, we created a vector of 0s and 1s, where 0s represented unmethylated CpGs and 1s represented methylated CpGs, proportionate to the observed methylation levels in the old samples. We randomized the order of the elements within this vector using the sample function in R, effectively shuffling the unmethylated and methylated CpGs. The randomized vector was then transformed into an $n \times m$ matrix, where *n* represented the number of reads in the young group and *m* denoted the number of CpGs in a given MspI fragment. These synthetic “random-old” reads retained the same number of read fragments as the young group, the same mean ccMet values as the old group, and reflected the same extent of random methylation as indicated by the ΔccMet values (i.e. ccMet level difference = old samples’ ccMet – young samples’ ccMet). To visualize ccMet patterns, we generated tiling plots using the methylation score matrices from the original data and the random-old models.

### Quantification of DNA methylation heterogeneity

We calculated the heterogeneity score (Hs) from DNA methylation patterns of individual cells in a sample using Hamming distances and Shannon entropy [54]. A matrix of DNA methylation data ($r$) was prepared for each *Msp*I locus, where rows correspond to individual cells and columns represent CpG sites. We denoted the methylated sites as 1 and the unmethylated sites as 0. A weight vector *w* accounts for the number of covered CpG sites in each cell pair. The heterogeneity score $Hs\ ( r )$ is computed as


\begin{eqnarray*}
Hs\ \left( r \right)\ = \ \left( {\Sigma \ wi\ *\ Di\ /\ \Sigma \ wi} \right)\ *\ \left( {\Sigma \ wi\ *\ Si\ /\ \Sigma \ wi} \right),
\end{eqnarray*}

where $Di$ is the normalized Hamming distance and $Si$ is the joint Shannon Entropy between a pair of cells, and $wi$ is the weight corresponding to the number of covered CpGs for that cell pair. We implemented this method in the R environment to compute a weighted score for heterogeneity for a given *Msp*I locus.

### CD4+ T-cell isolation

To isolate PBMCs, spleens of 3-, 22-, and 23-month-old C57BL6/J mice (*n* = 3 each) were pressed through a cell strainer (Corning) and washed with phosphate-buffered saline (PBS) containing 10% bovine serum albumin. Negative isolation of CD4+ T cells from the isolated PBMCs was performed using the Dynabeads Untouched Mouse CD4 Cells Kit (Invitrogen) [51]. Briefly, heat-inactivated fetal bovine serum and the Antibody Mix targeting various lineage markers excluding CD4 (provided in the isolation kit) were added to the PBMCs in the isolation buffer provided. The cell suspension was incubated with frequent tilting at 4°C for 20 min and then centrifuged at 350 × *g* for 8 min at 4°C. After the PBMCs were washed and resuspended in the isolation buffer, they were incubated with pre-washed depletion Dynabeads for 15 min at room temperature with gentle tilting and rotation. Finally, the tube containing the bead-bound cells was placed in the magnet and the supernatant containing the untouched CD4+ T cells was carefully collected. The remaining cells bound to Dynabeads were suspended and lysed for genomic DNA extraction.

### Production of TET1-KO and TET3-KO HAP1 cells

Small guide RNAs (sgRNAs) we used in specifically targeting the *TET1* gene are 5′-GTTGCCCGAGAATGTCGGCT-3′ (sgTET1-1) and 5′-AGCTCCATCACTATATGCTT-3′ (sgTET1-2). sgRNAs used for *TET3* gene targeting are 5′-CGCTCCCCTGTACAAGCGAC-3′ (sgTET3-1) and 5′-AGAACCAGGTAACGGGCCCT-3′ (sgTET3-2). The DNA oligonucleotides for these sgRNAs were mixed and annealed with equal amounts (100 pmol each) of complementary oligonucleotides prior to phosphorylation with T4 kinase (NEB) at 37°C for 30 min. The resulting short DNA was ligated into the pSpCas9 (BB)-2A-Puro plasmid (PX459; Addgene) to produce PX459-sgTET1s and PX459-sgTET3s using T4 DNA ligase (NEB) for 2 h at 16°C. Correct cloning was confirmed by sequencing. To generate knockout (KO) cell lines, the PX459-sgTET1 and PX459-sgTET3 plasmids were transfected into the control HAP1 cells using Lipofectamin^TM^ 3000 transfection reagent (Thermo). Forty-eight hours after transfection, the cells were cultured in puromycin (1 µg/ml) for 7 days. The resistant cells were then separated into single cells, diluted, and further cultured individually in 96-well plates. After clonal expansion, these cells were genotyped by PCR followed by Sanger sequencing and also experimentally validated by dot blot analysis to confirm the successful KO of the *TET1* and *TET3* genes. Primer sets used in PCR genotyping are as follows: 5′-CTCTCTCTTAAGATTGTGCTCCA-3′ and 5′-GCATTTAATGAAGACCTGCACT-3′ for *TET1*; 5′-CCCACGATGCTTCAGGAATG-3′ and 5′-ACTTCTGGACTAGGCTCTTCC-3′ for *TET3*. The PCR program constitutes an initial denaturation at 95°C for 5 min, followed by 35 cycles of denaturation at 95°C for 30 s, annealing at 55°C for 30 s, and extension at 72°C for 1 min with a final elongation step at 72°C for 5 min.

### Real-time PCR and western blotting

Total RNAs were extracted from spleens using RNeasy Plus Mini Kit (Qiagen) according to the manufacturer’s instructions. In total, 2 μg of total RNA was treated with 10 U of recombinant DNase I (Takara) for 30 min at 37°C, followed by ethanol precipitation. DNase I-treated total RNA was reverse-transcribed using 50 pmol of random hexamers and 200 U of SuperScript III Reverse Transcriptase (Thermo Fisher Scientific). A quantitative real-time PCR (qPCR) was performed with 10 ng of complementary DNA and 2× SYBR Green Fast PCR Master Mix (Applied Biosystems) on QuantStudio 3 Real-Time PCR System (Applied Biosystems). Primer sets used in PCR genotyping are as follows: 5′-GCATTTAGATGCCGGTTGAG-3′ and 5′-GGCAAGCAATGTCTACGTCC-3′ for *Adgrb3*; 5′-TGGAGCGCTATGATGAACAG-3′ and 5′-GTGGCTGTGCTCATCTGTGT-3′ for *Dio3*; 5′-CCTTGCATTACGACTACCTCCAG-3′ and 5′-TGCTGTCATCCAGCAGACTCTC-3′ for *P3h2*; 5′-GAGAAGTGGCACTGCTTGCTGA-3′ and 5′-ACCAACTGCTCCTCTGACATGC-3′ for *Spats2*; and 5′-CTCGGGGATGTAAAGGTGAA-3′ and 5′-CTTTGGCCCACACCATAAAG-3′ for *Sox17*. Ct values of target genes were normalized by those of GAPDH transcripts using the –ΔΔCt method to calculate fold change.

For western blotting, liver and spleen tissues from 4- and 25-month-old C57BL6/J mice were homogenized using a mortar and pestle in pre-chilled lysis buffer [150 mM NaCl, 1% Triton X-100, 0.5% sodium deoxycholate, 0.1% sodium dodecyl sulfate, 50 mM Tris–HCl (pH 7.4)] containing protease and phosphatase inhibitors (Sigma–Aldrich). The homogenates were incubated on a rotator at 4°C overnight and then centrifuged at 13 500 × *g* for 10 min at 4°C. Protein concentration was determined using the Bio-Rad Protein Assay according to the manufacturer’s instructions, with absorbance measured at 562 nm. For sodium dodecyl sulfate–polyacrylamide gel electrophoresis, 50 μg of protein lysate was resolved on a 7% polyacrylamide gel and transferred to nitrocellulose membranes at 150 mA for 1.5 h. The membranes were blocked with 5% nonfat dry milk and 0.1% Tween-20 in PBS for 2 h at room temperature, then incubated overnight at 4°C with primary antibodies (1:500–1:1000 in blocking buffer). After washing, membranes were incubated with HRP-conjugated secondary antibodies (1:1000–1:2000) for 2 h at room temperature. Antibodies used were anti-TET1 (GTX124207, GeneTex), anti-TET3 (GTX121453, Genetex), and anti-GAPDH (#2118, Cell Signaling). Protein bands were visualized using an ECL chemiluminescence detection system (Cytiva), with GAPDH serving as a loading control.

## Results

### Characteristics of ccMets in aged spleen

**Figure 1.**
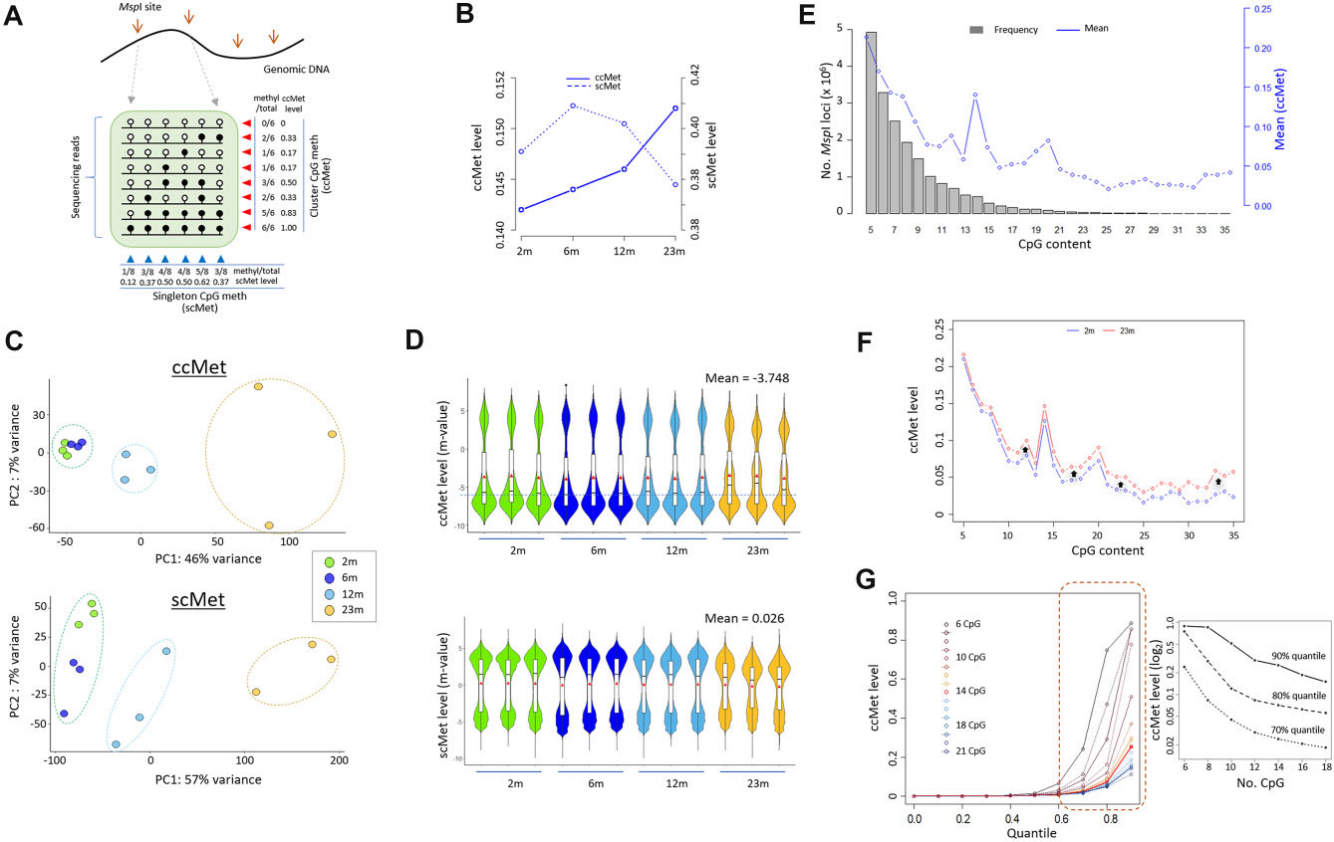
Characterization of DNA methylation statuses at spatially clustered CpG sites. (**A**) Definitions of ccMet and scMet. Methylation levels of genomic *Msp*I fragments (RRBS reads) are determined at either singleton- or cluster-CpG level. (**B**) Comparison of age-associated global methylation changes between scMet and ccMet analysis. Briefly, 2-, 6-, 12-, and 23-month-old mouse spleens (2m, 6m, 12m, and 23m, respectively; *n* = 3 for each group) were used in the RRBS analysis. (**C**) PCA using ccMet (top) and scMet levels (bottom). (**D**) Violin plots showing the ccMet-level (top) and scMet-level (bottom) distributions. The sample mean methylation levels are indicated by a dot on each plot. (**E**) Frequencies of *Msp*I fragments with given numbers of CpG sites (bars) and their mean ccMet levels (line). (**F**) Consistent increase of ccMet levels with age across all CpG intervals. (**G**) Dependence of ccMet levels and distributions on their CpG density. The higher quantiles consistently decreased with increasing CpG content.

We recently reported the genomic region-based global methylation states of singleton CpGs from RRBS of *Msp*I-digested mouse spleen DNA at 2, 6, 12, and 23 months of age (2m, 6m, 12m, and 23m, respectively; *n* = 3 for each group; [17]). To investigate novel aspects of age-associated changes in DNA methylation, an alternative analytical approach was employed, enabling the study of methylation at clustered CpGs using the same dataset (Fig. 1A). The level of ccMet was determined by calculating the mean ratio of methylated to total CpGs within sequencing fragments from a single *Msp*I locus. We selected 50 689 CpG clusters (CpG ≥ 5 and depth ≥ 10 in each sample per locus) for further analysis. The loci exhibited an average ccMet level of 0.146, which increased with age and was significantly lower than the corresponding scMet level of 0.395, which decreased over time (Fig. 1B). The higher scMet levels may be attributable to the presence of highly methylated remote CpGs located outside CGIs [17]. PCA for ccMet levels showed tighter clustering and lower variability among samples, except for the 23m group, compared with scMet levels (Fig. 1C). Violin plots revealed a distinct asymmetric dumbbell-shaped distribution of ccMets, with a larger lower lobe (Fig. 1D). Notably, the medians of the 23m samples exhibited markedly higher values, indicating an increase in methylation in numerous low-methylated clustered CpGs.

We observed a negative correlation between the number of *Msp*I loci and CpG content [ranging from 5 to 35; Spearman’s rank correlation coefficient (R) = −0.81; Fig. 1E). ccMet levels also decreased with increasing CpG counts (R = −0.82). When accounting for locus length, a similar correlation was observed (R = −0.77). The data demonstrated that methylation levels increased with age across all CpG intervals (Fig. 1F). Re-plotting ccMet levels of *Msp*I loci with 6–21 CpGs revealed that the upper quantile ccMet levels exhibited a decline as CpG content increased (Fig. 1G), underscoring the existence of distinct ccMet characteristics that are influenced by CpG density.

### ccMet analysis reveals heterogeneous cell distribution of blanket methylation

**Figure 2.**
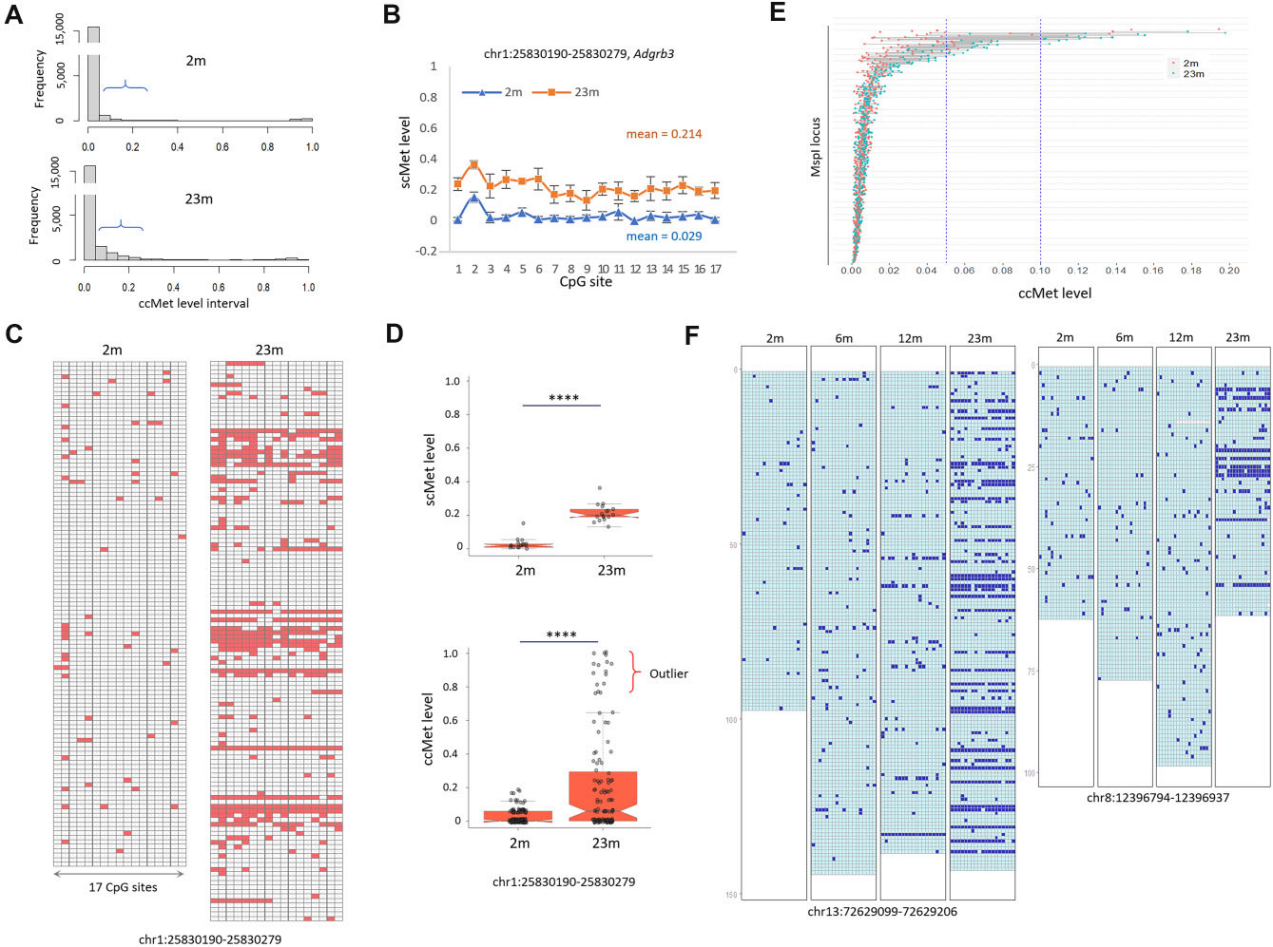
Discovery of BM from ccMet analysis in aged spleen. (**A**) Frequency of ccMet levels in 2m and 23m spleen samples. Brackets indicate the methylation intervals showing a significant increase in ccMet levels in the 23m group. (**B**) Relatively uniform scMet levels among 17 CpG sites in the *Adgrb3* locus (chr1:25 830 190–25 830 279). (**C**) ccMet tiling plots showing methylation profiles at the same locus as in panel (B). Filled and empty rectangles indicate methylated and unmethylated CpGs, respectively. Methylation tilings were generated using 118 (2m) and 130 reads (23m). (**D**) Distributions of scMet and (top) ccMet levels (bottom) at the same locus as in panel (B) in the 2m and 23m groups. A substantial variance among the ccMet levels is observed in the 23m group. The asterisk indicates a significant difference (*P* < .0001, Wilcoxon test). The bracket denotes the outlier values of ccMet. (**E**) Pairing of 2m and 23m ccMet levels for the same MspI loci. Reference lines for ccMet levels of 0.02 and 0.05 are indicated. (**F**) ccMet profiles for two *Msp*I loci (chr13:72 629 099–72 629 206 and chr8:12 396 794–12 396 937) in different age groups. Empty and filled rectangles designate unmethylated and methylated CpGs, respectively.

A comparative analysis was conducted on the 2m and 23m samples, representing the young and old age groups. The old group displayed increased methylation at a number of *Msp*I loci, most notably in low ccMet intervals (Fig. 2A). A locus comprising 17 CpGs in the *Adgrb3* gene promoter was randomly selected. Regarding scMet, both age groups exhibited relatively uniform hypermethylation across this locus (Fig. 2B), which was typical of other randomly selected loci (Supplementary Fig. S1). In contrast, the ccMet analysis of the same locus in the 23m samples revealed a near-dichotomous methylation status, with levels either very high or very low (Fig. 2C). Notably, a considerable proportion of the fragments derived from this locus exhibited ccMet levels above 0.75, which were classified as outliers (12% of 130; Fig. 2D). Nonparametric statistical tests revealed significant differences in ccMet distribution between the age groups (*P* < .0001, Mann–Whitney *U*-test and Kolmogorov–Smirnov test), indicating that the changes in ccMet status with age are neither random nor continuous (see below).

Globally, the majority of genomic *Msp*I loci exhibited ccMet levels below 0.1, with ccMet differences between the 2m and 23m (ΔccMets = 23m − 2m) primarily ranging from 0.01 to 0.02 (Fig. 2E). More sites were hypermethylated than hypomethylated (*n* = 19 450 versus 12 300). Furthermore, significant hypermethylation (ΔccMets > 0.1) was observed with a frequency 14.5 times greater than that of significant hypomethylation (ΔccMets < 0.1; *n* = 2019 versus 140). Low-methylation CpG clusters (LMCs) were selected based on the criteria of a ccMet level of <0.1 at 2 months of age and a ΔccMet value >0.1, which was deemed an appropriate threshold for identifying discernible hypermethylation events. A total of 1523 LMCs were selected for monitoring methylation changes across different age groups. Fig. 2F illustrates two representative *Msp*I loci where the methylation profiles demonstrated minimal change at 6 months. By 12 months, sporadic changes occurred at individual alleles or cells, and by 23 months, a significant fraction of cells had acquired full methylation at these CpG clusters, a phenomenon we term “blanket methylation” (BM). We also analyzed a public dataset (GSE213628; [20]), which includes PBMC RRBS data from 4- and 24-month-old mice, and confirmed the presence of BMs in the PBMC samples from older mice (Supplementary Fig. S2). A random, probabilistic increase in methylation levels with age would be incompatible with this pattern of methylation change. The discontinuous and nonprobabilistic nature of the observed methylation changes suggests that they are not solely due to random mechanisms.

### Specific CpG clusters undergo methylation changes in a nonrandom manner

**Figure 3.**
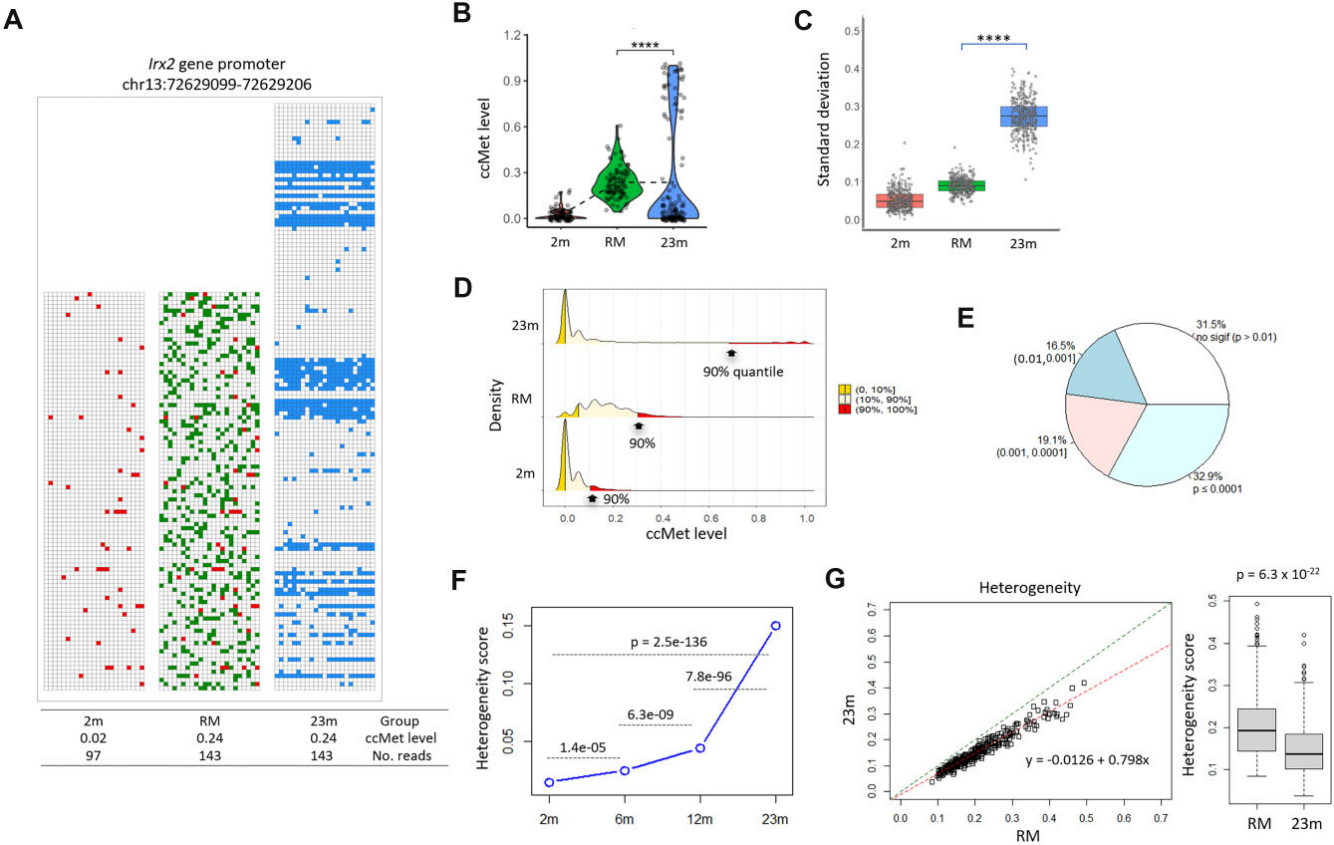
Nonrandom methylation changes at clustered CpGs. (**A**) Comparison of methylation states between 23m and RM. Blank and colored rectangles designate unmethylated and methylated CpGs. In the RM sample, two categories of methylated CpGs—those inherited from the 2m sample and newly acquired (randomly methylated) sites—are visually distinguished in the figure. Unmethylated CpGs remain unfilled. The coordinate of the locus is indicated. (**B**) Distribution of ccMet levels in the RM and 23m groups. The same locus as in panel (A) was analyzed. The group mean values are connected by a dotted line. (**C**) Standard deviations (SDs) of the ccMet levels of the 354 loci in the sample groups. The asterisks in panels (B) and (C) indicate a significant difference (*P* < .0001). (**D**) The bottom and top quantile ranges of ccMet levels (0–10% and 90–100%) are marked with distinct shading. A thick arrow designates the ccMet level of 90% quantile in each group. (**E**) Summary of statistical test (Wilcoxon test) of the 354 loci. In total, 8091 MspI loci were analyzed after filtering based on stringent criteria (≥15 CpGs and ≥50 depths per locus). Among them, 3082 loci were hypomethylated, while 5009 were hypermethylated. The majority exhibited minimal or no changes in ccMet, clustering in the low ccMet range (0.00–0.05) throughout aging, as seen in spleen data (see Fig. 2E). However, 354 super-hypermethylated loci met the BM selection criteria (<0.1 ccMet at 2m and ΔccMet ≥ 0.1). These thresholds were applied to enhance the detection of BM by identifying loci with clear full-methylation transitions over time. Percent values indicate the proportion of *Msp*I loci with *P*-values in the corresponding *P*-value category, as tested by the Wilcoxon method. (**F**) Age-associated increase of Hs. (**G**) Scatter plot for Hs. Hs were determined for the 354 loci and compared between the RM and 23m groups. The lines represent the 1:1 reference and the fitted regression line for the scores. The box plot shows the distribution of Hs.

To evaluate the hypothesis that age-associated methylation changes occur in a stochastic fashion, synthetic random-model (RM) fragments were generated for each *Msp*I locus. The fragments were designed to maintain the read fragment count and the original methylation state of the 2m group (point 1), to match the mean ccMet level of the 23m group (point 2), and to acquire random methylation equivalent to the ΔccMet level observed between the two groups (point 3). Of the LMCs, 354 CpG-dense loci, each containing 15 or more CpGs, were analyzed in order to establish a clear picture of the methylation changes across a wide range of CpG positions. Fig. 3A provides an example of the ccMet status at the *Irx2* gene promoter. Despite exhibiting the same mean ccMet level (0.24), the RM and 23m fragments exhibited distinct methylation profiles, indicating that the observed change in the 23m group was not random. The violin plot for this locus revealed a dumbbell-shaped distribution in the 23m group, with a distinct clustering of highly methylated fragments at the upper end, which distinguished the 23m samples from the 2m and RM samples (Fig. 3B). Additional violin plots and methylation tiling plots for selected loci are presented in the Supplementary Fig. S3A and B.

A statistical evaluation was conducted on the mean ccMet levels of all 354 loci. The SDs in the 23m group were found to be significantly higher than those in the RM group (0.27 versus 0.09; Fig. 3C). Similar outcomes were observed for the interquartile range (IQR = 75%–25%), a metric for the statistical dispersion of the data, and the coefficient of variation (CV) (Supplementary Fig. S3C). The density plot over deciles revealed a significant disparity in the distribution of ccMet levels. The 90th percentile of the ccMet levels for the 23m fragments was found to be significantly higher than that observed in the RM group (Fig. 3D). Lastly, two-third (68.5%) of the loci demonstrated differences in ccMet levels between the 23m and RM samples (*P* < .01, Wilcoxon test; Fig. 3E).

An Hs was calculated using Hamming distances and Shannon entropy formulas between all possible combinations of fragment pairs within the same sample [54]. The selected 354 loci exhibited a pronounced increase in Hs with age, indicative of substantial alterations in methylation patterns at these loci (Fig. 3F). A comparison of the RM and 23m groups revealed a significant reduction in Hs values in the latter (0.20 ± 0.08 versus 0.15 ± 0.06; *P* < .0001; Fig. 3G). The result could be interpreted as follows: While the heterogeneity among *Msp*I fragments increases with age, the heterogeneity in the 23m group does not reach as high a level as that of the RM group, probably due to the emergence of a population of cells with homogeneously methylated fragments as BM in the 23m group. In addition, the Hs values in other *Msp*I loci (CpG ≥ 10, *n* = 15 559) also exhibited a tendency toward an increase with age (Supplementary Fig. S4A), albeit at a slower rate than that observed in the selected 354 loci (Supplementary Fig. S4B). Supplementary Fig. S4C presents a comparison of individual Hs values between ages. In conclusion, these results demonstrate that the 23m group significantly differed in ccMet statuses from the RM group and, furthermore, age-associated methylation changes occurred in clusters, exhibiting a mosaic appearance between cells, as well as sporadically at the singleton CpG level.

### Age-associated BM in diverse tissues

**Figure 4.**
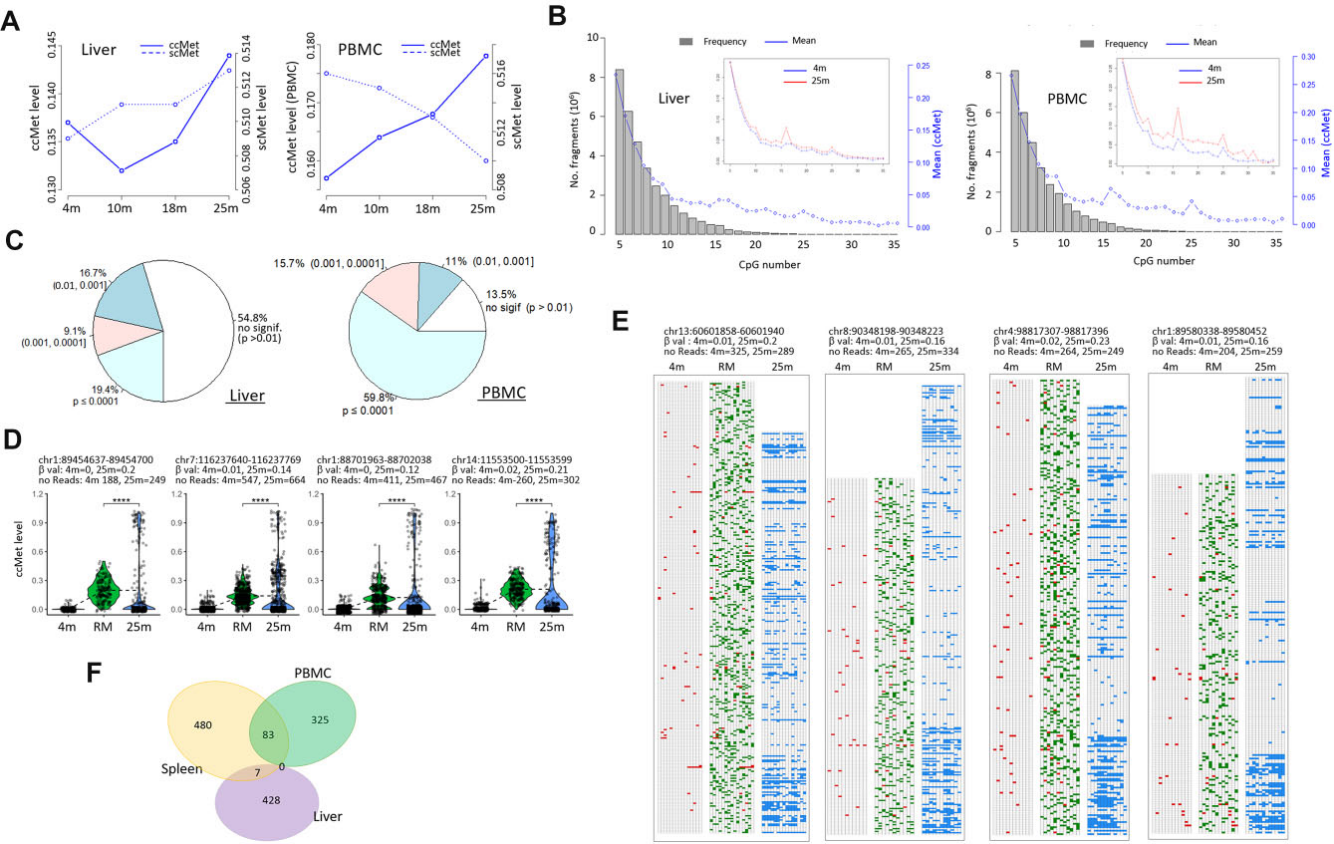
Age-associated emergence of BM in liver and blood samples. (**A**) Comparison of age-associated global methylation changes between scMet (dotted line) and ccMet analysis (solid line). Briefly, 4-, 10-, 18-, and 25-month-old mouse liver and PBMC samples (4m, 10m, 18m, and 25m, respectively; *n* = 5 for each group) were used in the RRBS and DNA methylation phasing analysis. (**B**) Frequencies of *Msp*I fragments with given numbers of CpG sites (bars) and their mean ccMet levels (line). Insets show an increase in ccMet levels with age across all CpG intervals. (**C**) Differences of ccMet levels between the RM and 25m samples. In total, 263 (liver) and 252 (PBMC) *Msp*I loci were selected based on stringent criteria (≥10 CpGs for liver or ≥15 CpGs for PBMC, ≥50 depths per locus, <0.1 ccMet levels at 4 months, and ΔccMet thresholds of ≥0.05 for liver or ≥0.1 for PBMC between 4m and 25m). Percent values indicate the proportion of *Msp*I loci with *P*-values in the corresponding *P*-value category, as tested by the Wilcoxon method. (**D**) Violin plots showing distributions of ccMet levels of four representative *Msp*I loci in the RM and 23m groups. The asterisk indicates a significant difference (*P* < .0001, Wilcoxon test). (**E**) Appearance of BM in 25m PBMC samples but not in the matching RM group. The head in each tiling plot contains information about the coordinate of the given cluster, ccMet levels (β-val), and the number of reads. (**F**) Identifying common *Msp*I loci whose ccMet levels are significantly different between the 25m and RM groups (*P* < .01). Three-way Venn diagrams for spleen (*n* = 567 in total), liver (435), and PBMC samples (408) are shown.

Whether cell-heterogeneous BM is present in other aged tissues was investigated. We analyzed liver and PBMCs from four age groups: 4m, 10m, 18m, and 25m (*n* = 5 in each group). The ccMet levels in liver and PBMC samples showed hypermethylation trends with age (Fig. 4A), and strong negative correlations with CpG number (R = −0.979 in the liver and −0.940 in PBMC; Fig. 4B). Similarly to the spleen, ccMet levels rose with age across CpG intervals in both tissues (Fig. 4B insets). We selected 263 (liver) and 252 (PBMC) loci on the basis of ccMet < 0.1 at 4 months and ΔccMets ≥ 0.05 (liver) or ≥0.1 (PBMC) between 4m and 25m samples. The lower ΔccMet threshold for liver selection was due to its subtle methylation changes in this tissue between the age groups. With regard to the distribution of ccMet levels among the selected loci, a significant difference was observed between the 25m and synthetic RM samples (Supplementary Fig. S5A). Particularly in PBMCs, 86% of the loci exhibited a significant difference (*P* < .01, Wilcoxon test; Fig. 4C). A notable BM was evident in both violin and tiling plots for PBMCs (Fig. 4D and E). Although the liver exhibited inherently low ccMet levels and less dynamic methylation changes with age, a subset of loci demonstrated age-associated BM (Supplementary Fig. S5B and C).

We next assessed whether the loci displaying disparate ccMet levels between the 25m and RM groups (*P* < .01 and SD > 0.25 for PBMC and spleen and *P* < .05 without SD filtering for liver) were shared across tissues. The genomic coordinates of these loci were extracted and compared among the spleen, PBMC, and liver. A total of 567, 408, and 435 loci were obtained from spleen, PBMC, and liver, respectively, with no common locus found (Fig. 4F). Spleen and PBMC shared 83 loci, whereas other pairs shared few. This suggests that while BM is a common phenomenon in aged tissues, its frequency and distribution vary by tissue.

### Individual variability in BM and its correlation with aging

Upon examination of each of the old spleen samples individually, it was observed that the presence of BM varied at the 10 randomly selected loci (Supplementary Fig. S6A). The mean fraction of BM fragments in the samples was 7.2%, 16.1%, and 11.9%, respectively (Supplementary Fig. S6B). The majority of these loci were associated with gene promoters, and the expression of genes that are expressed in the spleen (*Adgrb3, P3h2*, and *Spats2*) was found to be decreased (Supplementary Fig. S6C). In contrast, *Sox17* and *Dio3/Dio3-os* genes, whose CGIs were either distant from the promoter or encompassed the entirety of the genic region, showed increased expression.

**Figure 5.**
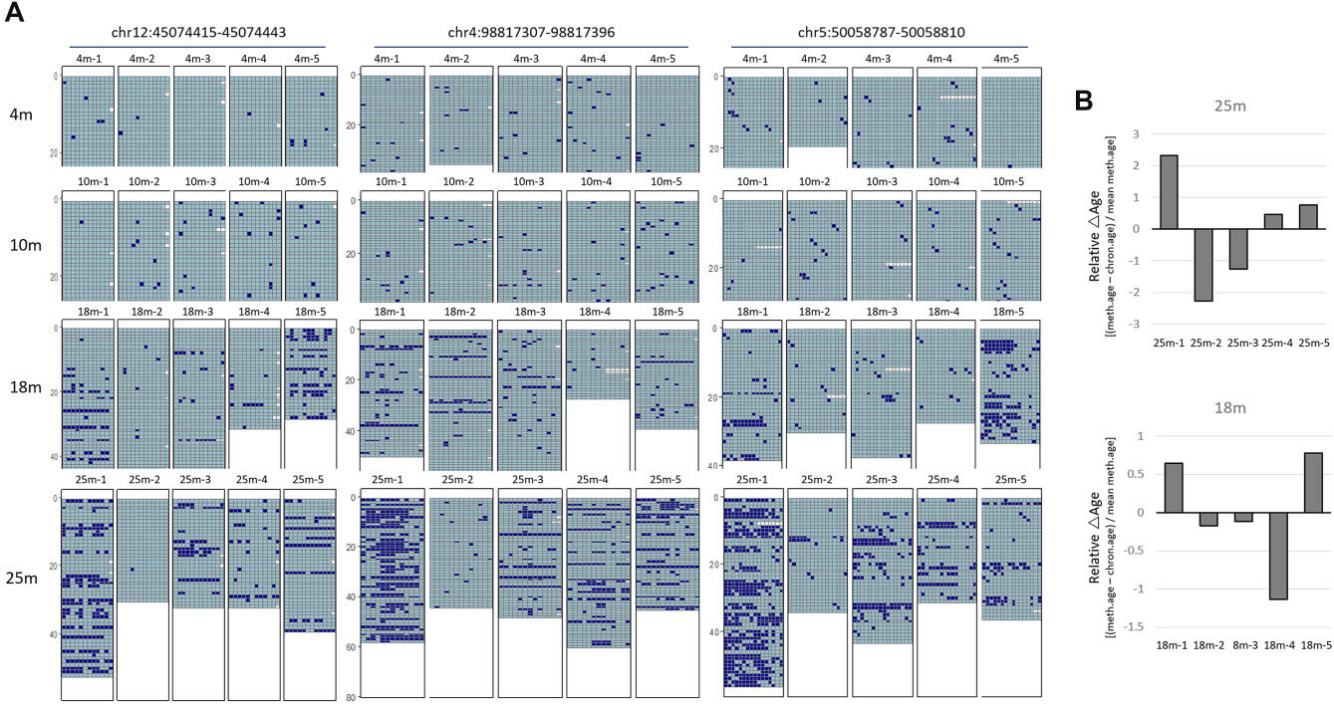
Higher frequencies of BM are associated with accelerated aging. (**A**) Methylation profiles of three representative *Msp*I loci in individual PBMC samples. Each age group has five samples as indicated by number. (**B**) Prediction of methylation age. Relative age differences (△age) of individual PBMC samples are calculated as follows: [(methylation age − chronological age)/mean methylation age of a given group]. The methylation ages of individual samples were obtained using our PBMC clock CpG sets. The *y*-axis values represent the months.

PBMCs displayed greater individual variability than spleens (Fig. 5A). For instance, while comparable frequencies of BM were observed in PBMC samples 25m-3, 25m-4, and 25m-5, a notable proportion was identified in sample 25m-1, and it was minimal in 25m-2 across all examined loci (of the 16 loci examined, only 3 are shown). The methylation ages, as predicted using our own PBMC methylation clock (unpublished data), revealed the highest age in sample 25m-1 and the lowest in 25m-2, showing a 4-month gap (Fig. 5B). Samples 18m-1 and 18m-5 exhibited greater methylation levels than the others, suggesting an advanced age as assessed by the methylation clock. Conversely, sample 18–4 exhibited reduced methylation levels, which align with its younger estimated methylation age. A similar trend in methylation-age differences was observed when applying Thompson’s clock [55] (Supplementary Fig. S7). This suggests that the occurrence of BM differs between individuals and tissues, with its frequency correlating with accelerated aging. Furthermore, as observed above (Fig. 2B), the uniform increase in scMet levels across the 23m spleen samples may be attributed to the presence of BM in a subset of cells, collectively resulting in elevated mean scMet levels.

### BM is an intrinsic aspect of the aging process

**Figure 6.**
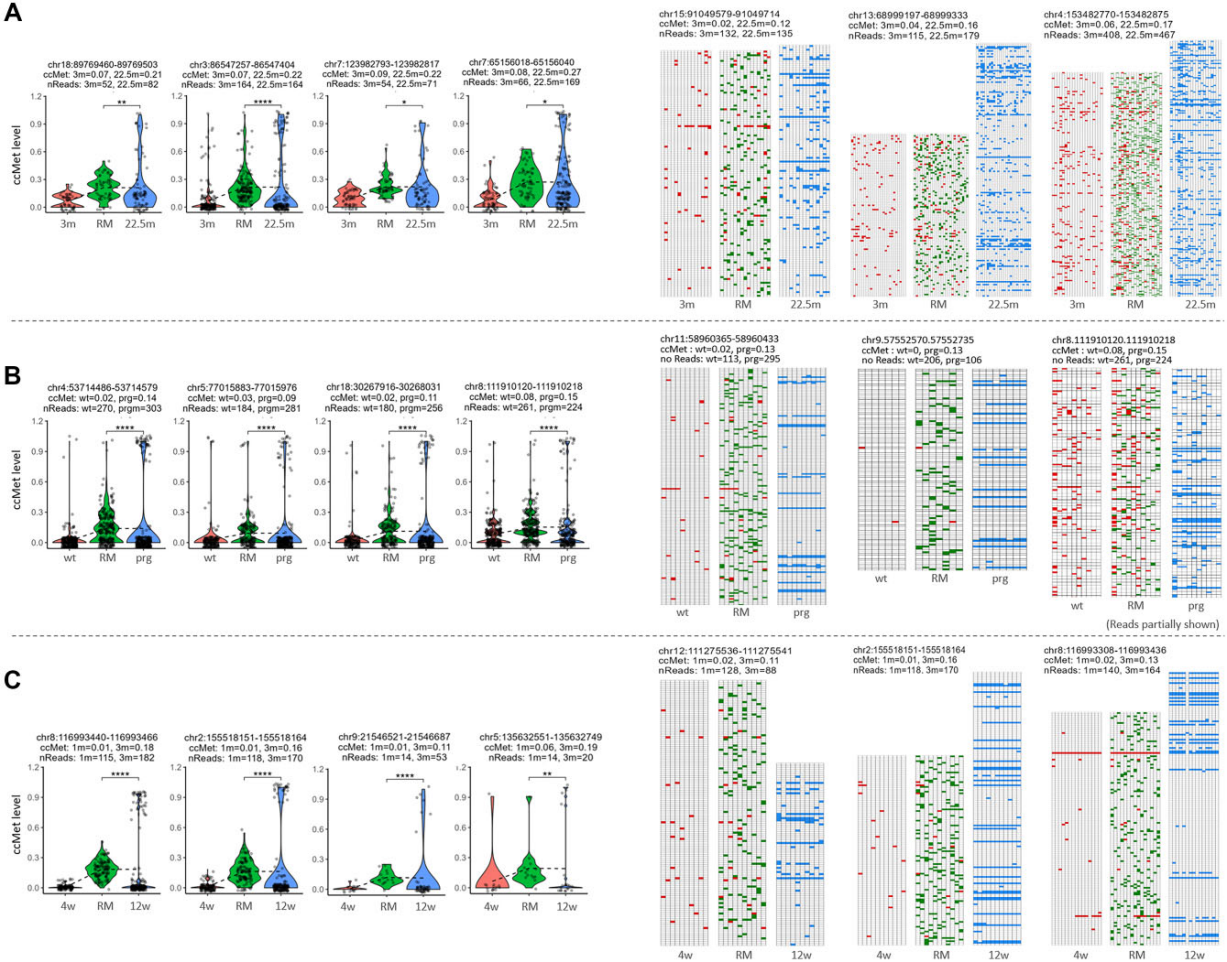
The emergence of BM is intrinsic and aging-accelerated. (**A**) ccMet levels of CD4-positive T cells in the 3m, RM, and 22.5m groups. 102 *Msp*I loci were selected using the criteria: CpGs ≥ 15 and depth ≥ 100 per locus, ccMet levels < 0.1 at 3 months, and △ccMets ≥ 0.1. (**B**) ccMet levels of liver tissues in 4-month-old wild-type (wt; C57BL6/J) and *Zmpste24^−/−^* mice (prg). The tiling plots only partially represent the methylation profiles due to their large quantity. (**C**) ccMet levels of liver tissues in 4-week-old (4w) and 12-week-old (12w) R6/2 HD model mice. In panels (B) and (C), 86 and 109 loci were selected using the criteria: ccMet levels of controls < 0.1, and ΔccMets > 0.05. *P*-values are denoted (*P < .05; **P < .01; ***P < .001; ****P < .0001). In panels (A)–(C), representative tiling methylation plots are shown on the right.

BM may be derived from cells of disparate tissue origins or from expanding a previously modest population of cells. To further explore this phenomenon, CD4+ T cells were isolated from PBMCs of 3-month-old (*n* = 4) and 22.5-month-old (*n* = 5) mice. Following the application of the aforementioned criteria (see above), 102 loci were selected. Seventy-four percent of the loci demonstrated statistically significant differences (*P* < .05) in the ccMet level distribution between the RM and 22.5m samples. Fig. 6A shows the occurrence of BM in CD4+ T cells from the 22.5-month-old group, as evidenced by both violin and tiling plots. From the remaining non-CD4+ cells, 231 loci were selected, exhibiting comparable results to those observed in CD4+ T cells, both statistically (with 71% showing a difference in ccMet levels) and in tiling profiles (Supplementary Fig. S8). These findings suggest that BM is an intrinsic aspect of the aging process. However, the results do not totally exclude the possibility that BM results from external cell infiltration or age-associated change in cell composition.

To further investigate BM in a model of accelerated aging, we examined the livers of 4-month-old *Zmpste24^−/−^* mice [56]. Zmpste24 deficiency results in progeroid phenotypes in both mice and humans, with affected mice succumbing at ∼20 weeks [57]. We performed RRBS using genomic DNA from Zmpste24^−/−^ mice (*n* = 3) and compared their DNA methylation patterns with age-matched wt controls. Of the 86 loci selected (ccMet < 0.1 in wt controls and ΔccMets > 0.05), 63% exhibited significant differences (*P* < .05) in ccMet level distribution between the synthetic RM and the progeroid model samples (Fig. 6B). BM was observed in the *Zmpste24* KO livers, reinforcing the notion that BM is closely linked to aging and may be particularly prevalent in models of accelerated aging.

As previously reported [51, 58], accelerated aging was also observed in mice with an HD model. To assess the presence of BM in this model, we analyzed liver samples from HD mice. Given that R6/2 HD mice have a lifespan of 14–17 weeks and HD phenotypes manifest at ∼8 weeks of age [59], we utilized 4-week-old (*n* = 5) and 12-week-old (*n* = 6) R6/2 mice as the young and old groups, respectively. Among the 109 loci selected (ccMet < 0.1 at 4 weeks, and ΔccMets > 0.05), 64% exhibited significant differences (*P* < .05) between the synthetic RM and 12-week-old samples. A comparable level of BM was evident in the old group (Fig. 6C), with a frequency that exceeded that observed in the 25m normal liver samples (Fig. 4 and Supplementary Fig. S5). This result indicates that BM occurs more frequently in cells and tissues undergoing accelerated aging, suggesting that it may serve as a marker for aging.

### Age-related en bloc demethylation at highly methylated CpG clusters

We examined whether CpGs in high-methylation CpG clusters (HMCs), which stand in contrast to the aforementioned LMCs, underwent synchronous demethylation. Two highly methylated neighboring *Msp*I loci with relatively steady scMet and ΔscMet values among the clustered CpGs in 2m and 23m spleen samples were randomly selected (Supplementary Fig. S9A and B). Intriguingly, ccMet levels at these loci were predominantly concentrated at the extremes of the distribution (>75% or <25%) in both groups. The majority of the observed methylation changes occurred in these end quartiles, while the intermediate-quartile levels remained stable (Supplementary Fig. S9C).

The number of HMC loci was considerably smaller than that of LMC loci. Applying the cutoff conditions of ccMet > 0.7 at 2 months and ΔccMet < −0.1 yielded only 72 loci. A comparison of the ccMet levels of these loci between the synthetic RM and 23m groups revealed overall statistical similarity, with the exception of six loci (Supplementary Fig. S9D). This lack of difference may be attributed to the presence of a small subset of unmethylated or minimally methylated fragments that were already present in the 2m samples (see Supplementary Fig. S9A), which may have reduced the contrast in ccMet level distribution between the age groups. Nevertheless, several lines of evidence point to en bloc demethylation. First, the violin plots for the 23m group display a dumbbell shape, with the lower dumbbell being wider than that of the RM group (Supplementary Fig. S9E), indicating an increase in the proportion of unmethylated fragments. Secondly, the methylation profiles demonstrate the enrichment of methylation-clean fragments in the 23m group (Supplementary Fig. S9F). Finally, the SD and CV values of ccMet levels exhibited notable discrepancies between the RM and 23m groups (Supplementary Fig. S9G). Collectively, these findings suggest synchronous demethylation in clustered CpGs.

### Reduced TET1 and/or TET3 activity in aged tissues is related to the appearance of BM

**Figure 7.**
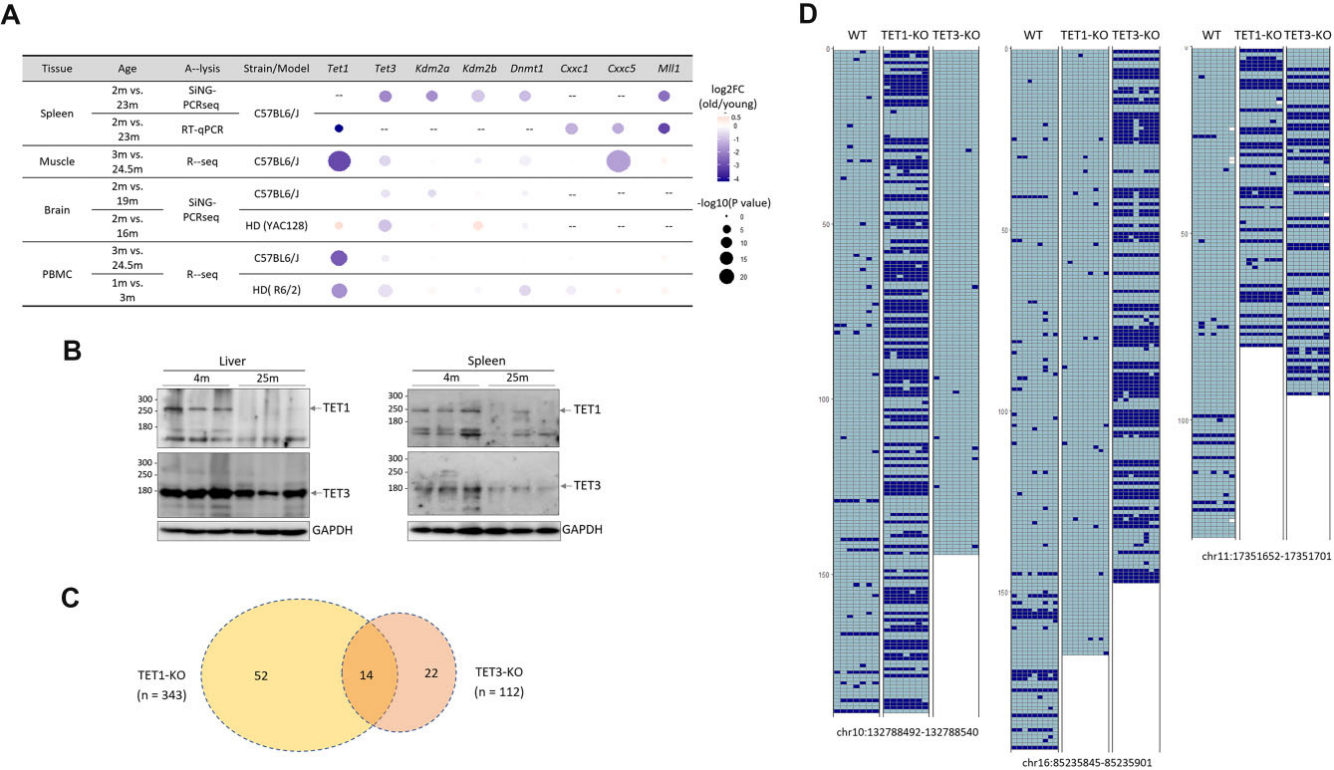
Reduced TET1 and/or TET3 activity in aged tissues contributes to the appearance of BM. (**A**) Comparison of expression levels of ZF-CxxC domain protein genes between young and old tissues in mice. Expression levels (*n* > 3) were assessed in various tissues (spleen, skeletal muscle, brain, and PBMC) of young and old mice using different platforms: SiNG-PCRseq for spleen and brain tissues, quantitative real-time PCR (RT-qPCR) for spleen, and RNAseq for skeletal muscle and PBMC samples. Tissue samples were collected from C57BL6/J mice at indicated ages or from HD model mice (YAC128 and R6/2). A gradient of black represents the fold change (old/young) in gene expression, and the statistical significance of the fold change is reflected in the dot size. “−” indicates missing data. (**B**) Western blotting results. Proteins were obtained from the livers and spleens of 4- and 25-month-old C57BL6/J mice (*n* = 3 each). (**C**) The number of CpG clusters displaying BM in TET1-KO and TET3-KO cells. The number (*n*) in the parenthesis indicates the total number of CpG clusters selected by the criteria: locus depth ≥ 100 per locus, ccMet levels < 0.1 in the control HAP1 cells, and △ccMets (KO − wt) ≥ 0.1. Of 343 and 112 selected clusters in the TET1-KO and TET3-KO cells, 66 and 36 exhibited BM, respectively. (**D**) Tiling plots for ccMet.

Unmethylated CpGs in CGIs are bound by cysteine-rich zinc-finger CxxC (ZF-CxxC) domain proteins, which include TET1, TET3, KDM2A, KDM2B, DNMT1, CFP1, and MLL1 [60, 61]. These ZF-CxxC proteins serve as nucleating factors at CGIs, establishing a protective architectural framework that inhibits *de novo* DNA methylation [62]. We investigated whether insufficient ZF-CxxC proteins are associated with the BM observed in aged tissues. By analyzing RNA sequencing (RNAseq) and spiking-in a neighbor genome for competitive PCR amplicon sequencing (SiNG-PCRseq) data [17] from various aged mouse tissues (spleen, muscle, brain, and PBMC) and verifying the findings with qPCR, a significant decrease in the expression of these genes was observed (Fig. 7A and Supplementary Fig. S10A). The expression of *Tet1* and *Tet3* was observed to decline frequently across aged tissues, which suggests that the protection of CGIs against erroneous methylation may be weakened in aged tissues. Supportively, the expression levels of *TET1* and *TET3* genes decline with age, concurrently with the 5hmc level, in blood cells, neural cells, and skin [42–45]. We confirmed the decreased expressions of TET1 and TET3 at the protein level. Western blotting results with liver and spleen tissue extracts from 4- and 25-month-old C57BL6/J mice (*n* = 3 each) showed that TET1 and TET3 levels markedly reduced in old samples (Fig. 7B).

To further explore this, we sought to determine whether the observed BM pattern in aged tissues also manifests in TET1- and TET3-depleted cells. *TET1* and *TET3* gene KOs were generated in near-haploid human HAP1 cell lines (Supplementary Fig. S10B–E), and the changes in methylation status across CGIs were assessed after RRBS (*n* = 4, 3, and 3 for wt, *TET1*-KO, and *TET3*-KO clones). Among the 121 158 *Msp*I clusters common to all three samples (locus depth ≥ 10), 343 clusters for *TET1*-KO and 112 for *TET3*-KO were selected based on the aforementioned criteria (ccMet levels < 0.1 in the controls, and ΔccMets ≥ 0.1). Blanket-type methylation patterns were observed in 66 (19.2%) *TET1*-KO clusters and 36 (32.1%) *TET3*-KO clusters, with 14 clusters shared between the two (Fig. 7C), indicating that CGIs are compartmentalized by TET1 and TET3 and are under differential control. The remaining clusters exhibited methylation increases in a random or CpG-dependent manner. Fig. 7D shows the differential occurrence of BM between *TET1*-KO and *TET3*-KO cells. The frequency of BM was higher in TET1-KO and *TET3*-KO cells compared with that observed in aged tissues. Therefore, the results support the hypothesis that the reduced TET1 and/or TET3 activity in aged tissues contributes to the appearance of BM.

## Discussion

At advanced ages, a small proportion of cells exhibit widespread, blanket-type methylation at specific CpG clusters that are scarcely methylated at young ages. Aging may progress with the expansion of this fraction of cells. This phenomenon merits further investigation to enhance our comprehension of the aging process. We employed unique molecular identifiers (UMI) to tag the captured *Msp*I-digested DNA fragments and remove PCR-duplicated read fragments, thereby ensuring that each RRBS fragment with a distinct UMI originates from a single allele of a cell. Thus, considering its partial presence within samples, BM can be understood as occurring in a subset of cells in a discrete manner, rather than changing uniformly in every cell and steadily over a lifetime. This is a novel finding of this study gained from a DNA methylation phasing study primarily focusing on allelic methylation changes at CpG clusters, which differs from the conventional approach of analyzing individual CpGs independently without consideration of spatial connections between CpGs.

With regard to the effect on gene expression, BM may decrease expression levels of highly active genes it associates with, while en bloc demethylation can increase expression of otherwise suppressed genes, resulting in noisy transcription. These bimodal alterations at extreme methylations and the resultant expression shifts comprise a tendency of “epigenetic and transcriptional drifts toward the mean” in cells of aged tissues [17, 58, 63]. Epigenetic drift is now considered to occur at the cellular, CpG cluster, and CpG site levels. There has been skepticism about whether minor and stochastic alterations at singleton CpG sites can have an impact on gene expression levels; it is now understood that a group of cells undergo comprehensive methylation changes at contiguous CpG-rich stretches in gene promoters. These changes appear to be more convincing and have practical implications for the transcriptional turn-on and -off of corresponding genes in aged tissues.

BM emerges in a locus-specific manner among tissue cells. The observation of BM in only a small fraction of cells gives rise to concerns about the potential infiltration of exogenous cells of disparate tissue origins, rather than an outcome of epigenome aging. However, this concern was mitigated by the observation of BM in purified CD4+ T cells as well as in whole PBMCs (Fig. 6A and Supplementary Fig. S8), indicating that these changes were not attributable to an inflammatory state or white blood cell redistribution. Similarly, if the emergence of BM in the spleen, PBMC, and liver had resulted from an increased immune cell population due to, e.g., age-associated chronic inflammation, a common set of CpG clusters with BM would have been expected in those tissues. However, no locus was identified to support this hypothesis (Fig. 4F). Taken together, these findings indicate that the phenomenon of BM is an intrinsic process of epigenome aging in aged cells.

One possible scenario for the emergence of BM is the loosening of the regulatory mechanism that safeguards CGIs from DNA methylation in aged cells. Although CGIs are typically resistant to methylation [64], the decline in ZF-CxxC protein activities that occurs with age may result in the exposure of CGIs to DNMTs, thereby leading to age-dependent methylation. This competitive dynamic between DNA methylating and methylation-blocking activities is postulated to regulate CGI methylation. For instance, KDM2B binds to unmethylated CpGs in CGIs, recruiting PRC1 and PRC2 complexes to protect these regions. However, with age-related degradation of these complexes, CGIs become more accessible to DNMTs, resulting in *de novo* methylation [11]. Furthermore, the downregulation of EZH2, the effector of the PRC2 complex, has been shown to trigger the onset of senescence [65]. When we looked for gene expressions of Ezh2 and its partner Eed in aged samples, no significant change was observed in PBMCs between the young and aged mice (*P* = .712 and .357, respectively). Even in the spleen (the same samples we used in this study), *Ezh2* gene expression was markedly increased in the aged group, while *Eed* exhibited minimal changes (Supplementary Fig. S10A). Therefore, it can be inferred that the involvement of PRCs is less likely. Another protective mechanism involves TET1, which is enriched at CGI boundaries to prevent the spread of methylation. In cells lacking TET1, methylation spreads to unmethylated CGIs [66]. Similarly, TETs and other CxxC domain proteins, which serve as scaffolding for the SET1/COMPASS complex, establish a high H3K4me3 chromatin environment [67] and consequently exclude DNMTs [68, 69]. TET3, which also contains a CxxC domain, may perform a function analogous to that of TET1. Our gene expression and western blot analyses revealed notable declines in TET1 and TET3 levels in aged tissues (Fig. 7A and B). In the *TET1* and *TET3* deletion experiments conducted in HAP1 cells, the resulting KO cells displayed BM in a subset of CpG clusters (Fig. 7D). Importantly, due to the near-haploid nature of the HAP1 cell line and the utilization of UMIs for deduplicating RRBS reads, each fragment is assumed to originate from a single cell. Interestingly, in these KO cells, BM occurred mosaically across the cell population, which resembled what we observed in our aged tissues. This finding may indicate that not all TET-governed CpG clusters undergo BM in every cell following TET KO. Alternatively, *de novo* DNMT activities may not efficiently methylate these loci. Either possibility can result in significant heterogeneity in the CGI methylation status and the transcriptional activity of the associated genes within a given TET-KO cell population. Similarly, cell-to-cell variability in TET expression in aged tissues could result in varied BM at CGI-associated promoters and enhancers, leading to heterogeneous expression of target genes. This epigenetic heterogeneity may inadvertently provide individual cells with distinct selective advantages, which could accelerate the aging process in some cells or drive others toward malignant transformation.

CpGs exhibiting methylation drift as a function of age may be regarded as prospective candidates for clock CpGs in age prediction. However, the majority of age-related CpGs display discontinuous and nonlinear changes in general, with only a limited number exhibiting a linear correlation with age. This raises the question of how methylation status at this specific set of clock-CpG loci changes with age and how the consistent change persists throughout the lifespan in the face of global stochastic changes in methylation. If, as is generally accepted, changes in methylation are stochastic, individual CpG sites are assumed to undergo such changes independently and randomly. This assumption is not applicable to the scenario in which the methylation of clock CpGs exhibits a uniform increase or decrease at a very low yet markedly stable rate throughout the lifespan. Our findings in this study provide a plausible explanation for the observed phenomenon: The number of cells exhibiting BM or en bloc demethylation increases with age, leading to a uniform rise in the methylation levels of individual CpGs in that CpG cluster. Notably, CpGs in close proximity to a clock CpG often display uniform methylation changes with age, despite exhibiting varying methylation levels (unpublished data). Therefore, the cluster- and cell-level methylation changes provide a valid explanation for the stable changes observed in clock CpGs over time.

Another fascinating question is why these peculiar cells arise in an aged cell population. We direct our attention to studies on CHIP (clonal haematopoiesis of indeterminate potential [70]), which is characterized by the expansion of haematopoietic stem cells bearing somatic mutations, most often in the DNMT3A and TET2 genes [71]. CHIP is a prevalent phenomenon that is becoming increasingly common with age. It is present in >10% of individuals over the age of 70 [72, 73] and its clinical implications are gradually becoming apparent [74]. Given that somatic mutations confer a selective advantage over those without, it seems probable that such a clonal expansion occurs in all tissues, as supported by studies of human skin [75] and the esophagus [76]. It is plausible that the distinctive cell population exhibiting local BMs may have originated from a CHIP-like event within the tissue, subsequently increasing in size with age. The differential methylation states reflect the disparate pattern of the CHIP-derived cells. It is conceivable that the process may be more rigorous in the PBMC and spleen than in the liver, which thus explains the higher frequency of BM in PBMCs and the lower frequency in liver tissues. A deeper understanding of the underlying event could provide new insights into age-related cellular and molecular changes and potentially identify targets for therapeutic interventions to mitigate adverse effects associated with aging.

A natural question arising from this study is whether the observed BM is a genome-wide phenomenon and whether whole genome bisulfite sequencing (WGBS) is necessary for confirmation. While WGBS provides comprehensive coverage, we argue that RRBS is sufficient to address this question, as it specifically targets CpG-dense regions such as CGIs, promoters, and regulatory elements that are critical for understanding methylation-mediated gene regulation. BM is unlikely to occur in CpG-sparse regions, which are less biologically relevant and beyond the scope of our analysis. Moreover, the ccMet metric enhances RRBS by accurately capturing methylation density in high CpG-density regions, yielding meaningful insights without requiring genome-wide data. Although WGBS could theoretically extend the analysis to CpG-sparse regions, its contribution would likely be minimal, given the biological focus of this study. Unlike WGBS, which randomly fragments the genome and does not target defined CpG clusters, RRBS offers consistent definitions of CpG clusters, enabling their precise comparison. Thus, RRBS provides a robust and targeted approach for investigating methylation changes, effectively meeting the study’s objectives without necessitating WGBS.

The differences observed between scMet and ccMet patterns highlight the complementary nature of these metrics and their distinct biological interpretations. scMet captures site-specific methylation changes at individual CpG sites, which can exhibit nonlinear variability influenced by stochastic or context-specific factors. In contrast, ccMet evaluates methylation density across CpG clusters, integrating signals from multiple CpG sites within a region and smoothing out site-specific fluctuations. This distinction explains the differing trends observed in Figs 1B and 4A. In the spleen (Fig. 1B), scMet exhibits a nonlinear pattern of increase, decrease, and re-increase, while ccMet shows a consistent upward trend. Similarly, in the liver (Fig. 4A), ccMet initially decreases at 10 months but increases at later timepoints, whereas scMet follows a more gradual increase. While DNA methylation exhibits interindividual variability, particularly at single CpG sites, the large-scale nature of our analysis minimizes its impact on the overall observed trends. The extensive number of CpG sites and CpG clusters analyzed ensures that the patterns observed in scMet and ccMet are driven by systematic biological changes rather than individual-level variation. Interindividual differences tend to be more prominent in local or small-scale methylation studies, whereas global or clustered analyses—such as those used in this study—reflect more robust age-associated trends. Furthermore, the use of biological replicates and statistical analyses mitigates potential individual variability, ensuring that the observed patterns are representative of broader biological phenomena. This integrative approach underscores the importance of analyzing methylation patterns from multiple perspectives to fully capture the complexity of age-associated methylation dynamics. By considering both site-specific and regional methylation changes, our study provides a comprehensive understanding of how DNA methylation evolves with age.

A potential concern regarding the use of R6/2 mice in this study is that HD models primarily exhibit neurological pathology and may not fully recapitulate systemic aging. While it is true that HD is fundamentally a neurodegenerative disorder, studies have demonstrated that HD models exhibit aging-like molecular characteristics, including epigenetic age acceleration in the brain [77] and systemic markers of aging, such as telomere shortening and chromatin accessibility shifts in blood cells [51, 58, 78–80]. These findings indicate that HD pathology extends beyond the nervous system and intersects with aging-related molecular processes. Although HD mice do not encapsulate all features of natural aging, their accelerated pathology provides a valuable system for investigating epigenetic alterations linked to aging-like changes. This approach contributes to a broader understanding of how aging-related molecular changes manifest in disease contexts, complementing studies conducted in traditional aging models.

HD models exhibit circadian rhythm dysfunction [81, 82] due to neurodegeneration in the suprachiasmatic nucleus and altered expression of key clock genes, including CLOCK, BMAL1, and PER1 [83–85]. While circadian disruptions can influence DNA methylation, these effects are typically gene-specific and oscillatory, differing from BM, which involves CpG cluster-wide full methylation shifts. The ccMet metric used in this study captures broad methylation density rather than periodic fluctuations, making it a robust measure for detecting aging-related methylation patterns. Given that BM was observed across multiple tissues, including livers and PBMCs, the findings suggest that BM is an epigenetic feature of aging rather than a direct consequence of circadian rhythm dysfunction.

In conclusion, this study provides valuable insights into the nature of age-related CpG methylation changes, with a particular focus on the emergence of BM at clustered CpGs. Our findings demonstrate that, as individuals age, there is a noticeable shift in methylation status, particularly within specific CpG clusters, where BM patterns emerge. These changes are not random but occur in a discrete and structured manner, indicating that age-related methylation follows a nonstochastic, mosaic pattern within cell populations, potentially influencing gene regulation. Moreover, the occurrence of BM across multiple tissues, including the spleen, liver, and PBMCs, suggests that BM is a widespread phenomenon linked to aging. However, its frequency and distribution vary between tissues, and significant interindividual variability exists, even among those of the same age. This points to the possibility that BM may correlate with individual rates of aging. Our findings also underscore the role of TET1 and TET3 proteins, whose reduced expressions in older tissues appear to contribute to the emergence of BM, as their absence leads to a higher frequency of this phenomenon. Finally, the discovery of BM as a potential biomarker for aging opens new avenues for understanding the molecular mechanisms of aging and age-related diseases. Further studies are warranted to explore the functional implications of these methylation changes and their relationship to age-related pathologies.

## Supplementary Material

gkaf354_Supplemental_File

## Data Availability

Sequencing data for spleen samples were deposited under GSE203289. Other sequencing data were deposited in K-BDS (Korea BioData Station, https://kbds.re.kr) with the accession ID KAP000001.
