# Supplementary material for: Emergence of CpG-cluster blanket methylation in aged tissues: a novel signature of epigenomic aging
Source: Nucleic Acids Res. 2025 May 10;53(9):gkaf354. doi: 10.1093/nar/gkaf354 (PMC12065108; doi:10.1093/nar/gkaf354)
Supplement: gkaf354_Supplemental_File [file gkaf354_supplemental_file.pdf]

Supplementary Figures and legends

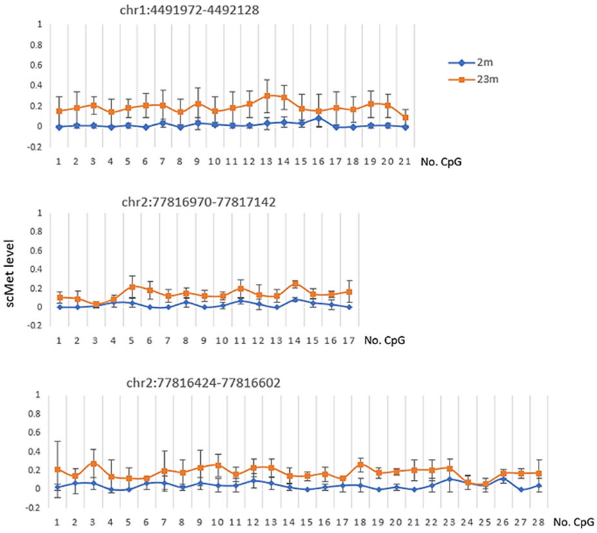

Figure S1 scMet changes in 2- and 23-month-old mouse spleens (2m, 23m, respectively; n = 3 for each group). The genomic coordinates for given *Msp*I loci are indicated.

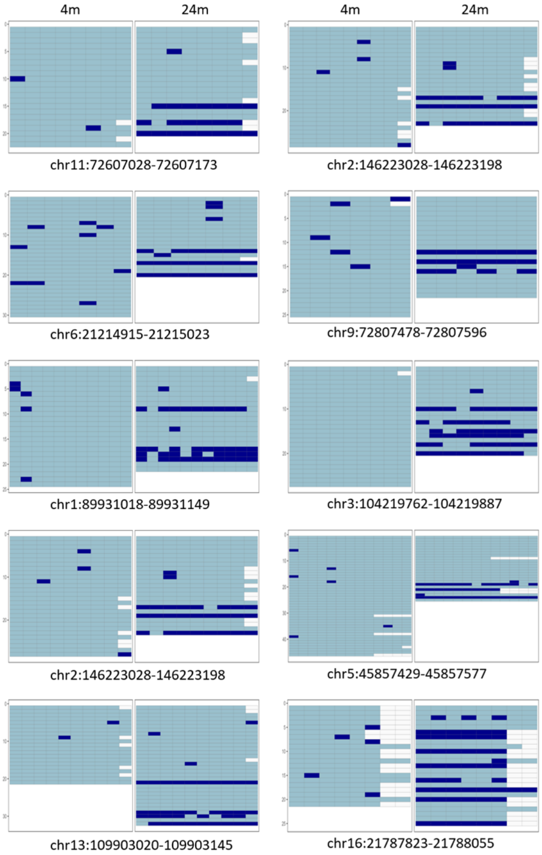

Figure S2 Presence of blanket methylation (BM) in PBMC samples from older mice.
RRBS Fastq files (SRR21627952, SRR21627953, SRR21627954, SRR21627955, SRR21627990, SRR21627991, SRR21627993, SRR21627994) were obtained from the NCBI GEO database (GSE213628) and processed using the Bismark tool for CpG methylation extraction and ccMet data reformatting. Due to limited data availability, samples from each age group (4 months and 24 months) were pooled for ccMet analysis. The coordinates of analyzed regions are displayed below the ccMet tiling profiles. Light and dark blue tiles indicate unmethylated and methylated CpGs, respectively.

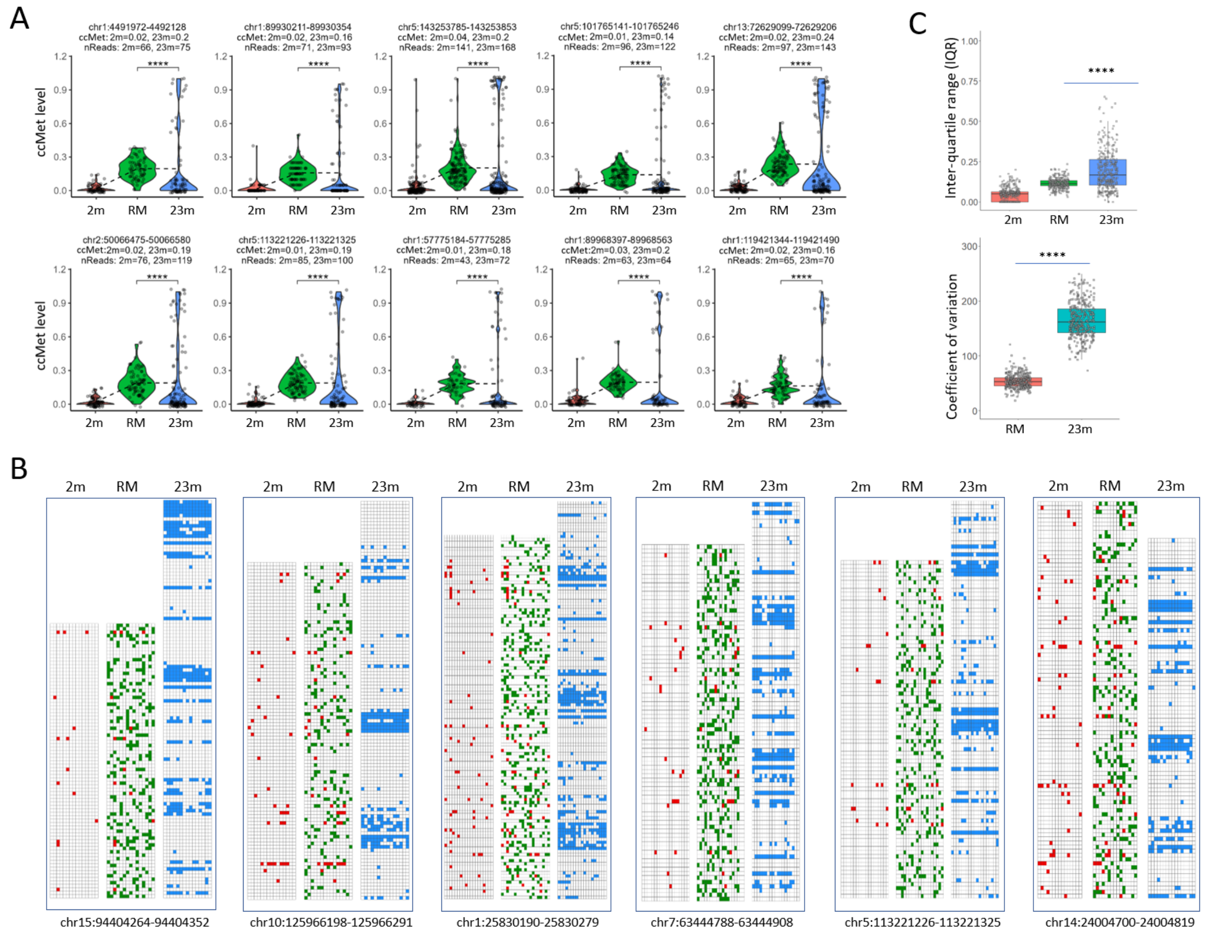

Figure S3 Plots of ccMets for selected *Msp*I loci showing a significant difference between RM and 23m groups.

A, Distribution of ccMet levels in the `rand' and 23m groups. The asterisk indicates a significant difference between groups ($p < 0.0001$, Wilcoxon test).

B, Tiling plots for methylation states comparing the 23m group and `rand' group. Blank and colored rectangles indicate unmethylated and methylated CpGs. In the "rand' sample, randomly methylated CpGs are indicated in green color in addition to truly methylated CpGs in red inherited from the 2m sample. The coordinate of the locus is indicated below.

C, Box plots of the ccMet levels of the 354 loci. In the top, the interquartile range (IQR = 75% – 25%), a measure of the statistical spread of the data, is significantly wider in the 23m than in the `rand' (0.17 vs. 0.11). In the bottom, the coefficient of variation (CV) differed significantly between the groups (161% vs. 53%).

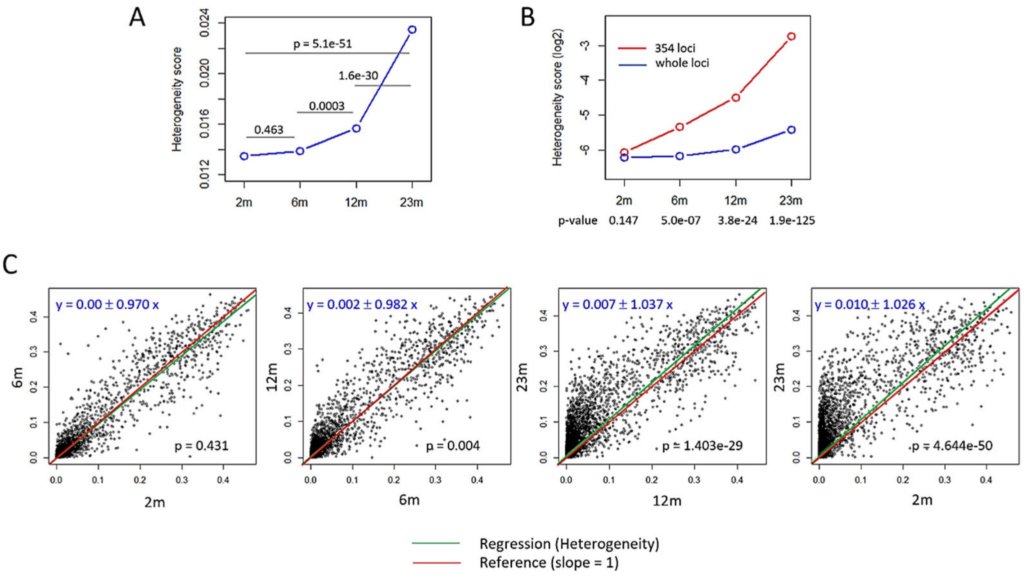

Figure S4 Heterogeneity scores in spleen samples.

A, Heterogeneity scores of the 15,559 *Msp*I loci (CpG >10) in spleen samples of different ages. P-values indicate differences between age samples.

B, Comparison of heterogeneity scores (in $\log_2$ scale) between the unselected (15,559 loci, blue) and selected 354 loci (red). P-values below indicate differences between the groups.

C, Comparison of heterogeneity scores between age groups. Heterogeneity scores of the 15,559 loci are compared. Green and red lines indicate the 1:1 reference and the regression line for the scores, respectively. The formula in blue indicates the slope and y-axis intercept of the regression line. P-values denote differences between given age groups.

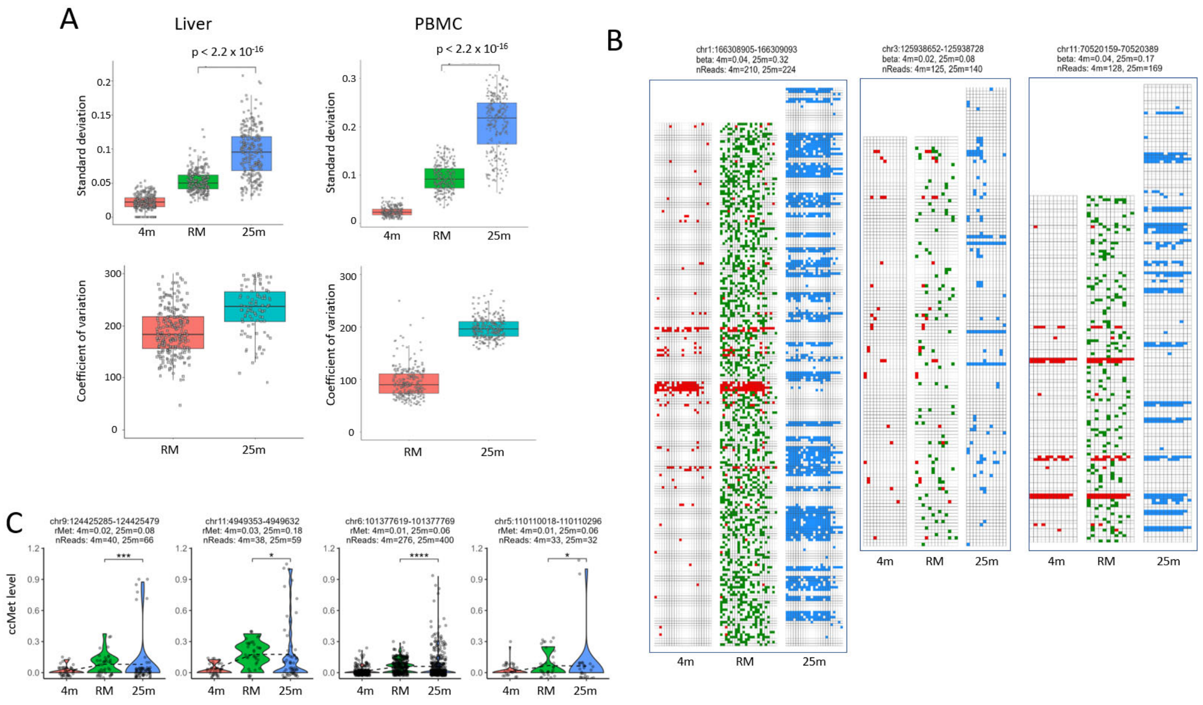

Figure S5 Analysis of ccMet levels in liver and PBMC samples.

A, Statistical test (Wilcoxon) of the 263 liver and 252 PBMC *Msp*I loci. The top and bottom graphs show the standard deviations and variation coefficients of ccMet levels of selected *Msp*I loci.

B, Methylation profiles for three *Msp*I loci in different age groups. The head in each tiling plot contains information about the coordinate of the given cluster, ccMet levels (beta), and the number of reads.

C, Distribution of ccMet levels in different age groups. *P*-values are indicated (*, $p < 0.05$; **, <0.01; ***, <0.001; ****, <0.0001).

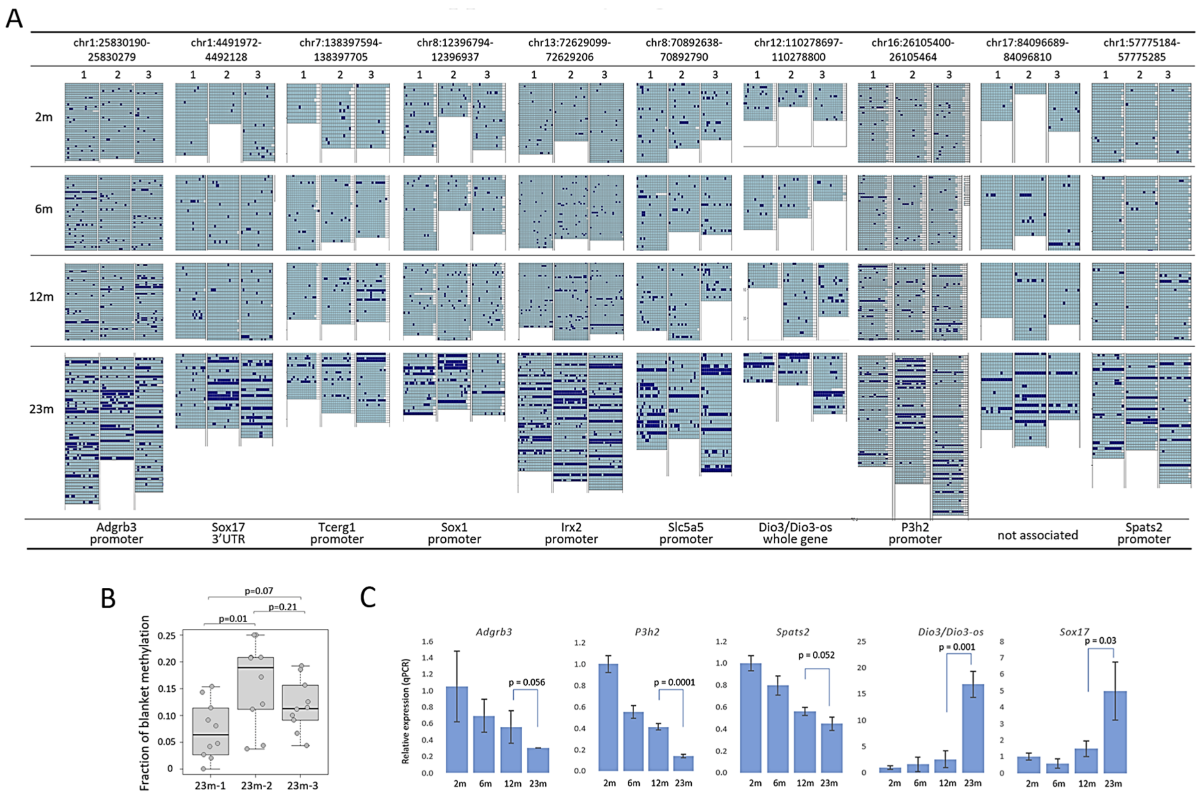

Figure S6 Variable frequencies of blanket methylation among different spleen samples.
A, The methylation state of ten *Msp*I loci is shown. Samples are designated by numbers (1 to 3). The associated genes are indicated below.
B, In box plot (bottom), each data point indicates the fraction of blanket methylation in a given *Msp*I locus of each 23m sample. P-values are shown (Paired-sample t-test).
C, Quantitiative real-time PCR for the associated genes.

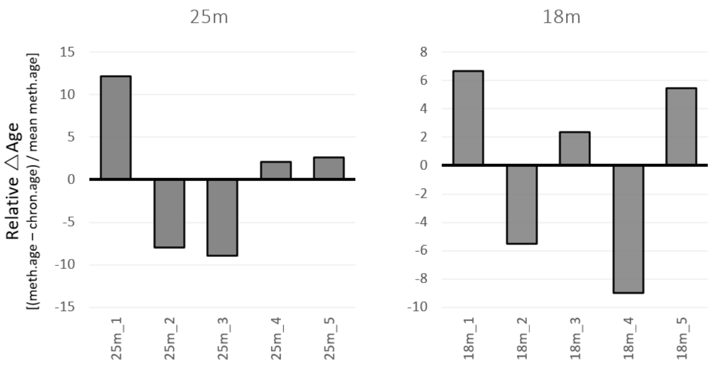

Figure S7 Prediction of methylation age based on Thompson's clock. Relative age differences (△age) of individual PBMC samples are calculated as follows: [(methylation age – chronological age) / mean methylation age of a given group]. The methylation ages of individual samples were obtained using our PBMC clock CpG sets. The y-axis values represent the months.

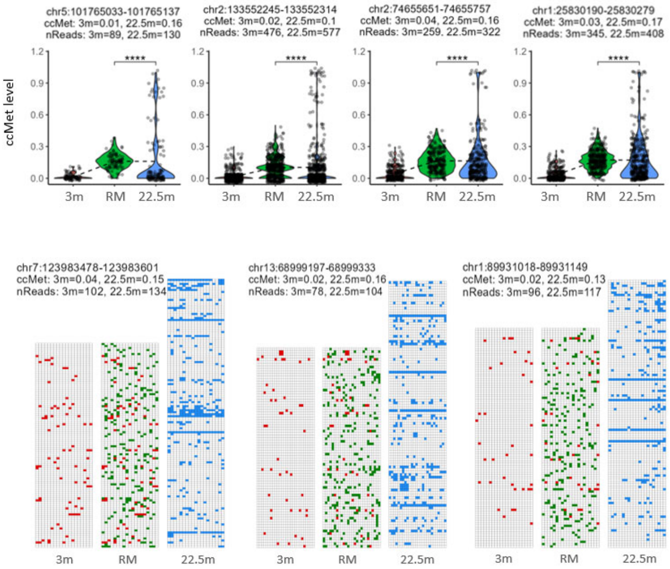

Figure S8 ccMet levels of CD4-negative PBMCs in the 3m, RM, and 22.5m groups. 102 *Msp*I loci were selected using the criteria: CpGs ≥ 15 and depth ≥ 100 per locus, ccMet levels < 0.1 at 3 months, and △ccMets ≥ 0.1. P-values are denoted (*, $p < 0.05$; **, <0.01; ***, <0.001; ****, <0.0001). Representative violin and tiling methylation plots are shown.

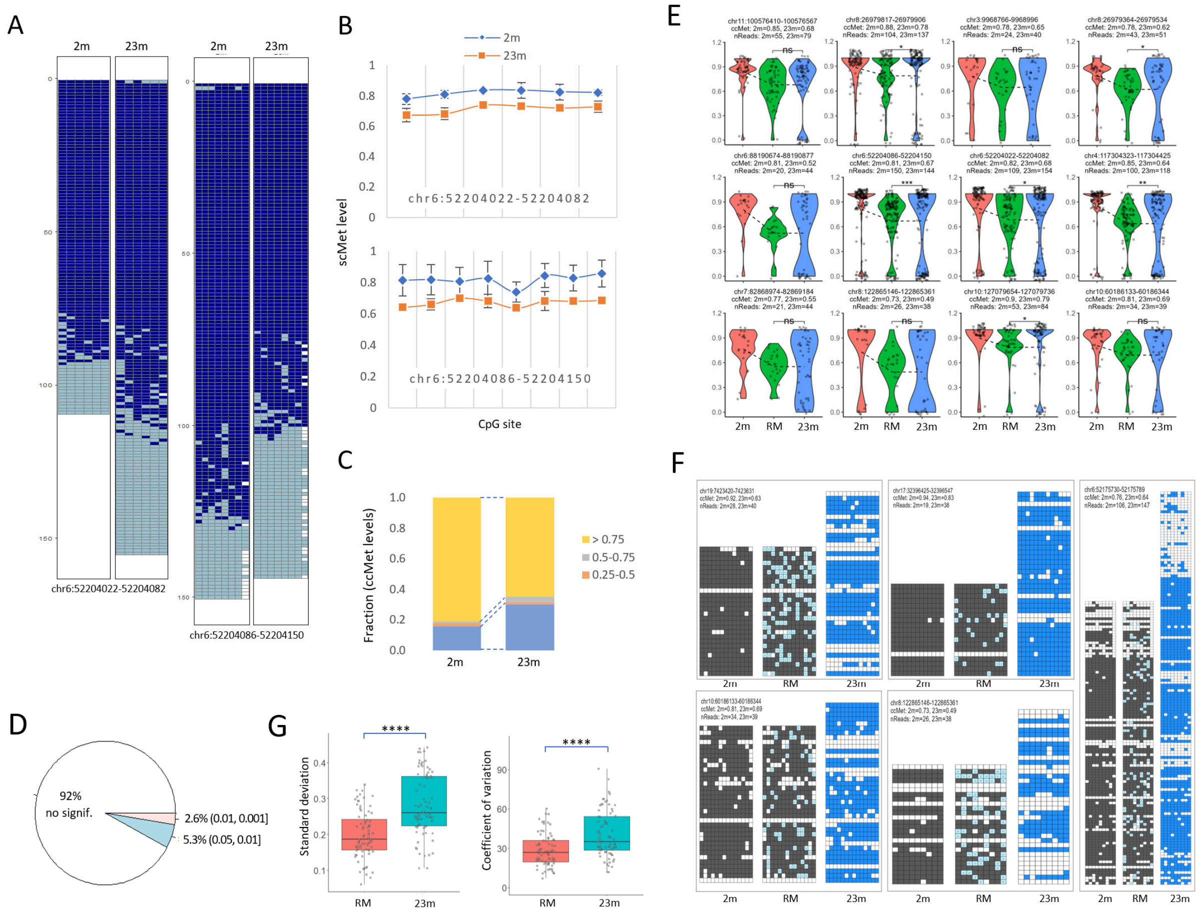

Figure S9 The occurrence of en bloc demethylation at high-methylation CpG-cluster loci
A, Tiling plots of methylation profiles in 2m and 23m spleens.
B, Comparisons of methylation levels of singleton CpGs at given *Msp*I locus between the 2m and 23m spleens.
C, Fractions of ccMet levels.
D, Summary of statistical test (Wilcoxon signed rank test) of 72 high-methylation CpG-cluster (HMC) loci. Percent value indicates the proportion of HMC loci with p-values in the corresponding p-value category.
E, Violin plots displaying a dumbbell shape for the 23m group. P-values are indicated (*, p <0.05; **, <0.01; ***, <0.001; ****, <0.0001; ns, >0.05). The head in each plot has information about the given cluster's coordinate, ccMet levels (β-val), and the number of reads.
F, The tiling methylation plots for hypomethylated loci. Blank and dark grey rectangles indicate unmethylated and methylated CpGs in the 2m group, respectively, and blue and sky-blue rectangles indicate methylated CpGs in the 23m group and `rand' group, respectively.
G, Statistical evaluation of ccMet levels of 72 HMC loci between the `rand' and 23m groups.

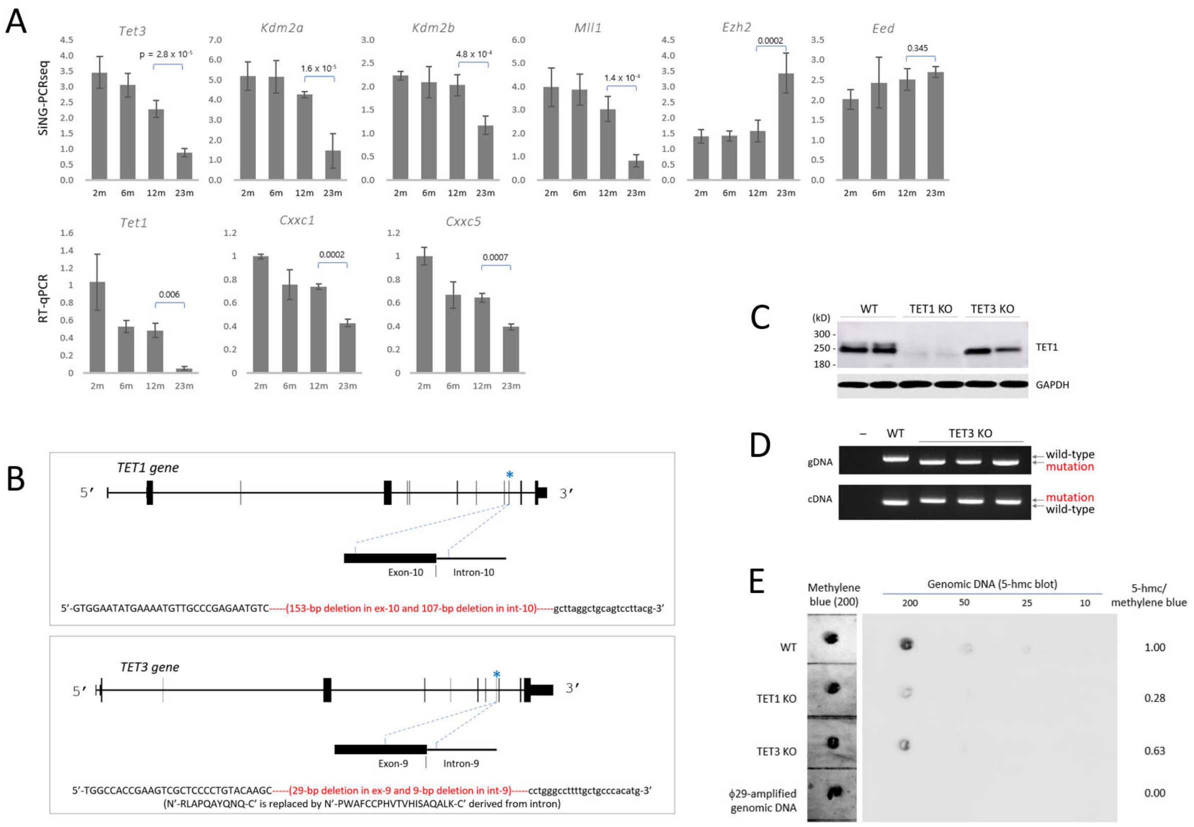

Figure S10 Comparisons of the ZF-CxxC domain protein genes' expression levels in the spleen samples and the generation of *TET1* and *TET3* knockout clones in HAP1 cell lines.

A, Gene expression levels were determined by SiNG-PCRseq or quantitative real-time PCR (RT-qPCR). P-values are indicated (*, p <0.05; **, <0.01; ***, <0.001; ****, <0.0001; ns, >0.05).

B, Genotyping by PCR. After clonal expansion, three clones for each gene were separately isolated and genotyped. These clones were identified to have the same mutation and thus assumed to have the same parental origin of cells in both TET1 and TET3 KO. The TET1 deletion mutation results in a frameshift and premature truncation of the protein. The TET3 deletion mutation replaces a segment of the catalytic domain (DSBH) with a sequence derived from the neighboring intron (N'-PWAFCCPHVTVHISAQALK-C').

C, Western blotting of TET1. Western blot result for TET3 was omitted because it barely discriminated between the wild-type and mutant TET3 proteins due to the similar size.

D, PCR analysis of the TET3 sequence. Primers used for gDNA PCR (indicated in blue arrows) and cDNA PCR (indicated in red arrows) are mapped in B. Primers used were 5'-CCCACGATGCTTCAGGAATG-3' and 5'-ACTTCTGGACTAGGCTCTTCC-3' for genomic DNA (gDNA), and 5'-CGGAAGAGTTTCCAGGACCT-3' and 5'-GGCACAGAAGTCCATGCAG-3' for complementary DNA (cDNA). PCR bands of different sizes, corresponding to wild-type cells and mutant clones, are indicated by arrows.

E, Dot blot analysis. Genomic DNAs were isolated, denatured in 0.1 M NaOH for 10 min at 95°C, and neutralized with an equal volume of 2 M ammonium acetate (NH_4OAc) on ice. Two ul of sample DNAs were spotted onto a nylon membrane (Cytiva) and allowed to air dry at room temperature before baking at 80 °C for 30 min. After blocking with 5 % skim milk in TBST for 1 hour at room temperature and incubated with anti-5-hmC antibody (39769, Active motif) overnight at 4 °C. The membrane was washed and incubated with a rabbit HRP-conjugated secondary antibody (7074, Cell Signaling Technology) for 1 h at room temperature before the dot signal was visualized using a chemiluminescent substrate (Cytiva). Phi29 DNA polymerase-amplified, methylation-erased whole genomic DNA was employed as a negative control. The equivalent amount of loaded DNA was confirmed by staining the membrane with 0.02% methylene blue. The intensity of the 5-hmC signal was quantified using ImageJ software.
